# The Role of Key Amino Acids in the Antimicrobial Mechanism of a Bacteriocin Model Revealed by Molecular Simulations

**DOI:** 10.1021/acs.jcim.1c00838

**Published:** 2021-12-07

**Authors:** Víctor L. Cruz, Javier Ramos, Javier Martinez-Salazar, Manuel Montalban-Lopez, Mercedes Maqueda

**Affiliations:** † BIOPHYM, Department of Macromolecular Physics, Instituto de Estructura de la Materia, IEM-CSIC, C/ Serrano 113 bis, Madrid 28006, Spain; ‡ Department of Microbiology, University of Granada, C/ Fuentenueva s/n, Granada 18071, Spain

## Abstract

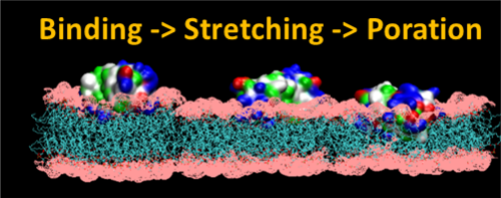

The AS-48 bacteriocin is a potent antimicrobial polypeptide with enhanced stability due to its circular sequence of peptidic bonds. The mechanism of biological action is still not well understood in spite of both the elucidation of the molecular structure some years ago and several experiments performed that yielded valuable information about the AS-48 bacterial membrane poration activity. In this work, we present a computational study at an atomistic scale to analyze the membrane disruption mechanism. The process is based on the two-stage model: (1) peptide binding to the bilayer surface and (2) membrane poration due to the surface tension exerted by the peptide. Indeed, the induced membrane tension mechanism is able to explain stable formation of pores leading to membrane disruption. The atomistic detail obtained from the simulations allows one to envisage the contribution of the different amino acids during the poration process. Clustering of cationic residues and hydrophobic interactions between peptide and lipids seem to be essential ingredients in the process. GLU amino acids have shown to enhance the membrane disrupting ability of the bacteriocin. TRP24–TRP24 interactions make also an important contribution in the initial stages of the poration mechanism. The detailed atomistic information obtained from the simulations can serve to better understand bacteriocin structural characteristics to design more potent antimicrobial therapies.

## Introduction

The bacteriocin AS-48 is a circular antimicrobial polypeptide produced by *Enterococcus faecalis*. AS-48 displays remarkable broad-spectrum activity against Gram-positive and some Gram-negative bacteria, including many antibiotic-resistant strains, having a remarkable potential in the food preservation field and in several clinical applications.^1−4^ Both the circularity of the AS-48 polypeptide chain and its amphipathicity are responsible for peptide stability at different temperature and pH conditions, as well as conferring resistance to some proteolytic processes.^1,5^ The primary target of AS-48 is the bacterial cell membrane, where it forms pores of around 0.7 nm in diameter, leading to membrane permeation not dependent on specific receptors or membrane potential.^1^ However, many details concerning this mechanism of action remain elusive, though some effort to improve its activity is being continuously done.^6^

**Scheme 1:**
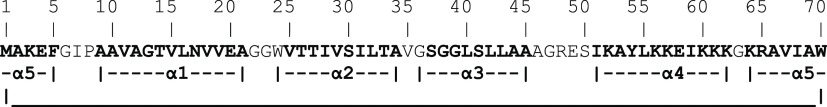
AS-48 Amino Acid Sequence Displaying the Localization of the α-Helices The head-to-tail linkage (M^1^-W^70^) is shown as a connecting line.

Scheme 1 shows the primary structure of the bacteriocin AS-48, a 70-residue α-helical peptide where a segregated distribution of positively charged and hydrophobic residues could be observed. NMR experiments in solution showed a globular arrangement of five α-helices.^7^ Further studies based on X-ray experiments were able to elucidate the crystal structures of AS-48 in different physicochemical environments.^8^ This crystallographic study determined two different dimer structures in hydrophilic (water solutions) and hydrophobic (membrane-bound state) environments. On the one hand, the soluble dimer in water (namely, DF-I) showed a structure similar to that described in the NMR analysis, i.e., the charged residues exposed to the solvent and surrounding the hydrophobic core amino acids. On the other hand, a dimeric form II (DF-II) was obtained under crystallization conditions that included the presence of a detergent to mimic the membrane-bound state. This DF-II presents a segregation of the hydrophilic residues, which can interact with solvent or charged groups on the membrane surface, from the hydrophobic ones that could interact with the hydrophobic core of the membranes. Based on these findings, the mechanism for bacteriocin action involves a transition from the water-soluble DF-I form to the membrane-bound state (DF-II form) upon membrane binding. The first step is the binding of the dimeric DF-I form to the bilayer surface by unspecific electrostatic interactions between the cationic residues of the peptide and the anionic phosphate groups of the lipids. In a subsequent step, the hydrophobic moiety of AS-48 is buried into the lipid membrane.^8^ Bioactivity studies performed by site-directed mutagenesis experiments support the fact that AS-48 activity could rely on the effective insertion of the bacteriocin into the membrane. On the one hand, it has been found that hydrophobic interactions between residues of both protomers should be weak enough to allow the mutual rotation of both protomers to change between different dimeric forms.^6^ Experimentally, the role of each of the four GLU residues (4, 20, 49, and 58) in the bacteriocin antimicrobial activity was studied by using separately genetically engineered GLU/ALA variants.^5^ A significant activity loss was found in each mutation except for the one corresponding to GLU20, which remains practically the same as the wild-type bacteriocin in accordance with its spatial position of the side chain of this residue. These findings together with another protein engineering performed on AS-48 allowed the conclusion that the wild-type AS-48 peptide should be “close to perfection”,^5^ although the detailed mechanism of action has not been fully proposed.

The AS-48 peptide has been proven to be selective to bacterial membranes over eukaryotic cells. The main difference between bacterial and eukaryotic cells is the high proportion of anionic lipids in the membranes of the former. Noteworthy, the cholesterol content in eukaryotic cell membranes can impede peptide insertion, too.^9^ This selectivity has been corroborated in coarse-grained molecular dynamics simulations by us, clearly showing the absence of interactions between the cationic peptide and the zwitterionic DPPC (dipalmitoylphosphatidylcholine) lipid bilayer, while it readily binds to an anioic DPPG (dipalmitoylphosphatidylglycerol) bilayer.^10^ Interestingly, some trypanosomatids with anionic phospholipids exposed to the external medium allow a privileged interaction with this type of strongly cationic peptide.^11^

The most common mechanism of membrane poration takes into account the bilayer stretching that is produced by peptide binding.^12^ Huang has proposed a two-state model for the action of antimicrobial peptides:^13^ (i) peptide bound to the bilayer surface and (ii) peptide insertion into the bilayer, when certain amounts of peptides are bound to the membrane. It has been shown that a progressive increment of the number of peptides bound to the bilayer surface yields an increment of the bilayer area per lipid until a plateau value is reached above a certain number of adsorbed peptides.^14−16^ The fractional area increment is linked to a fractional bilayer width decrement in accordance with the incompressibility of the lipid bilayer hydrocarbon phase. The overall result should be an increasing probability of membrane defects that can be recognized by the peptides to penetrate the bilayer forming pores.^17^

In this article, the antimicrobial AS-48 peptide action on bacterial membranes is studied using all-atomistic molecular dynamics (MD) simulations. An anionic lipid bilayer has been used as a model of the active site corresponding to the bacterial cytoplasmic membrane, as suggested in the literature.^18,19^

The work is divided into two different parts, namely, simulations of the binding process and exploration of the surface-tension-driven poration mechanism discussed above. As shown in Scheme 1, the AS-48 peptide possesses 10 positive (8 LYS and 2 ARG) and 4 negative (4 GLU) residues. At physiological pH, these residues remain charged, giving a net charge of +6. However, it is known that close to the bacterial membrane surfaces, the pH can strongly decrease,^1^ being sufficiently low to protonate the GLU to GLUH residues and, therefore, increasing the net charge to +10. To assess the effect of pH on the poration mechanism, we have performed MD simulations with both unprotonated (GLU) and protonated (GLUH) glutamic amino acids. In addition, the charged residues are mainly clustered in helices 4 and 5. It has been recently shown that an adequate level of charge clustering on antimicrobial peptides gives rise to an enhanced bactericidal effect.^20,21^

By analyzing the detailed information obtained from the atomistic models, it has been possible to explain different experimental observations on the AS-48 bactericidal bioactivity. Moreover, most of the results are also in close agreement with experiments and simulations performed with other antimicrobial systems.^22−30^

## Computational Methods

### Initial Structures

**Figure 1:**
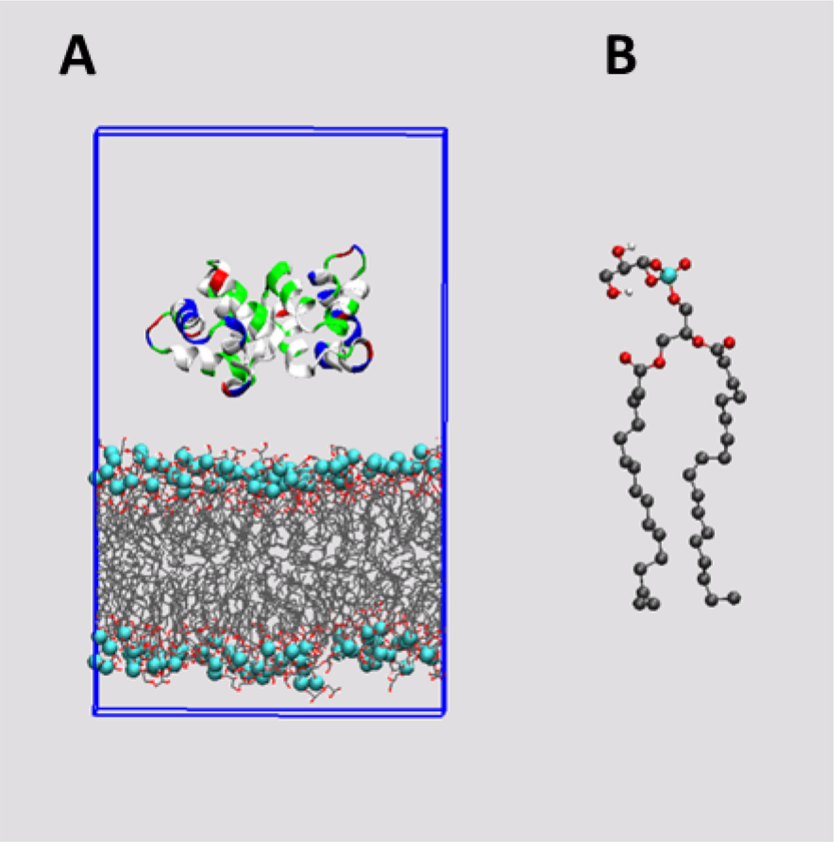
(A) Initial MD simulation box containing the AS-48 peptide and 128 POPG lipid bilayer. Lipid molecules are shown as lines and phosphorus on the lipids as cyan VDW balls. Water molecules and counterions are not shown. (B). CPK representation of the POPG molecule.

The initial structure of the AS-48 peptide was obtained from the PDB repository. The dimeric form DF-I was selected for this work (Figure 1A) (PDB-ID: 1O82).^8^ The lipid bilayer model was built based on the palmitoyl-oleyl-phosphatidyl-glycerol anionic molecule (POPG) (Figure 1B). We adopted the pre-equilibrated bilayer structure published by Kukol corresponding to a snapshot obtained after a 40 ns MD simulation.^31^ The bilayer was composed of 64 lipids of each chiral form, namely, D- or L-POPG, for a total of 128 POPG lipids. A layer of water molecules was added to enlarge the simulation box in order to accommodate the AS-48 dimer in the solvent phase. This was accomplished with the simulation tools available in the GROMACS software.^32^

The 128 POPG lipid bilayer system without the peptide described above was also considered to obtain a comparative behavior with the peptide-containing system. The composition of the system is shown in Table 1.

**Table 1:** Composition of Each Simulated System^a^^,^^b^

system	peptide number	POPG lipids	Na^+^ ions	water molecules
unprotonated	2	128	116	10,924
protonated	2	128	108	10,924
DPPG	n.a.	128	128	10,110

a n.a.: not applicable.

b GLU and GLUH reference to the peptides with unprotonated and protonated GLU residues, respectively.

The DF-I form of the AS-48 peptide was inserted in the middle of the solvent area, and water molecules within 2 Å of any peptide atom were deleted. Amounts of 116 and 108 Na^+^ ions were added to counterbalance the neutral and low pH systems, respectively (see Table 1 for a description of each system composition). This system should correspond to the highest peptide-to-lipid (P/L) ratio available for this bacteriocin, given its size and the limitations imposed by the minimum image convention that have to be adopted for those simulations. Nonetheless, the 1/64 P/L ratio may be acceptable because it is in line with the critical ratios experimentally determined, at which membrane poration is observed.^13,16^

### Simulation Methods

All the simulations and some analyses were performed using the GROMACS 2016 software suite.^33^ The GROMOS 56a4 force field^34^ was used for the AS-48 peptide. The SPC (Single Point Charge)^35^ model was adopted for water molecules. The lipids’ force field parameters and topology were taken from Kukol’s article.^31^

To study the two-step mechanism explained in the introduction, we have performed simulations in three different ensembles, semi-isotropic NPT and NVT for the binding step and NγT (surface-tension coupling ensemble) for the surface-tension-driven step. The semi-isotropic NPT-MD simulations have been accomplished using the Nose–Hoover thermostat^36,37^ with a time constant for coupling of 10 ps and a target temperature of 310 K for the equilibration and production stages. Semi-isotropic pressure coupling was accomplished following the Parrinello–Rahman scheme^38,39^ by coupling separately the bilayer plane dimensions (*X* and *Y*) and the normal plane to the bilayer (*Z* dimension). Values of 1 bar, 5 ps, and 4.5 × 10^–5^ bar^–1^ were taken for pressure, coupling constant, and compressibility, respectively.

Constant surface-tension simulations (NγT ensemble) are also available in GROMACS through a modification of the Berendsen pressure coupling scheme.^40^ In this case, it should be taken into account that the lipid bilayer presents two interfacial areas parallel to the *xy*-plane. The surface tension (γ) and the *z*-component of the pressure are coupled separately to the pressure bath using the same coupling constant (5 ps) and compressibility (4.5 × 10^–5^ bar^–1^) values as used in the NPT simulations. The z-component is coupled to a 1 bar pressure bath. The range of surface tensions considered in the present work was 50–70 mN/m. The range of temperatures was 310–350 K, using the same thermostat and conditions described above for the NPT ensemble.

A time step of 2 fs was selected for all molecular dynamics simulations.

The non-bonded interactions were calculated using the Verlet buffer cutoff scheme implemented in GROMACS using a van der Waals cutoff of 1.2 nm. Electrostatic interactions were calculated using the particle mesh Ewald (PME) method with a cutoff value of 1 nm, an interpolation order of 4, and a Fourier spacing of 0.12.^41^ Periodic boundary conditions (PBC) were applied at all of the three directions to remove the surface effect and to mimic the bulk state.

The initial models shown in Figure 1 were subjected to the following simulation protocol:

- Steepest descent energy minimization was used to relief steric hindrance in the initial solvent-peptide-lipid system.
- Ten nanosecond NPT molecular dynamics simulations with position restraints to non-water atoms were performed to equilibrate the solvent around the solute. After 10 ns, solvent distribution is equilibrated as shown in the solute–solvent interaction energies’ stabilization.
- In the case of semi-isotropic NPT systems, a long MD simulation of 600 ns at 310 K and 1 bar was performed for both models with unprotonated and protonated GLU residues.
- The final structure from the previous stage was used to start four replicas for each system in the NVT ensemble at 310 K.
- The study of the surface-driven step was achieved using NγT simulations at different surface tension values in the range of γ = 60–70 mN/m. The final structure from the previous stage was used to start three replicas for each system at temperatures and surface tensions of 310 K and 70 mN/m on one simulation set and 330 K and 60 mN/m on the other.

### Analysis Tools

Several Gromacs tools were used to analyze, among others, some H-bonds, minimum distance maps, and number of contacts. The evaluation of the number of contacts between protein and lipid groups was computed taking into account each contact individually.

SuAVE software tools were used to describe structural properties of the lipid bilayer.^42^ As a global parameter for the analysis, a set of 500 points per dimension was used to describe those bilayer characteristics.

Molecular graphics have been generated using the VMD package.^43^

## Results and Discussion

### Peptide Binding to the Lipid Bilayer

**Figure 2:**
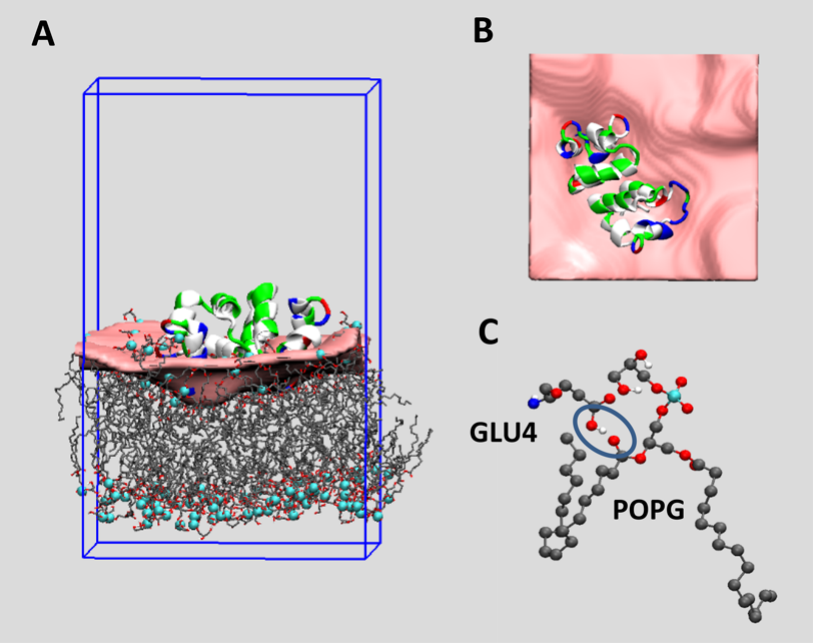
(A) Final snapshot of an NVT replica for the GLUH system. The peptide is represented as a cartoon colored according to the residue type (blue: cationic, red: anionic, green: polar, and white: apolar). The lipid bilayer molecules are represented as lines colored according the atom type. The lipid P atoms are shown as cyan VDW spheres. The upper layer surface corresponds to the grid obtained by SuAVE after fitting lipid P atoms of that monolayer. (B) Top view of the system depicted in panel A. Lipid molecules are not shown. (C) Representation of the H-bond between protonated GLU4 and one POPG lipid. The peptide residue and the lipid molecule are shown in CPK format. Encircled are the peptide GLU OH donor and the lipid carbonyl O acceptor.

The AS-48 bacteriocin is a cationic peptide with a net charge of +6 at neutral pH. However, in the range associated to its biological action, the decrease of pH found in the membrane interface can exceptionally protonate the four GLU residues, increasing the net charge from +6 to +10.^1^ Interestingly, it has been proposed that these four residues are situated in a plane that segregates the helices 4 and 5 (positively charged) from the hydrophobic moiety of AS-48 (helices 1, 2, and 3).^18^ Thus, the positive charges of LYS and ARG residues clustered in helices 4 and 5 are able to interact electrostatically with the surface of the negatively charged anionic lipid bilayer of bacteria, driving the peptide adsorption. The resulting structure is depicted in Figure 2 for the GLUH system (containing peptides with protonated residues). This figure includes a representation of the surface built by fitting the positions of the lipid P atoms using the SuAVE tools. To describe the bilayer surface, a set of 500 points per direction was used. A satisfactory RMSD (root-mean-square deviation between grid points and P atoms) value below 0.1 nm was obtained after the fitting. The top view that is shown in Figure 2B allows one to visualize the surface deformation produced by the peptide binding.

It can be observed that not all the cationic residues interact with the lipid phosphate groups, with some of them remaining in the water phase. It is interesting to observe that the GLUH peptides penetrate deeply the lipid bilayer due to the formation of H-bonds between the GLU proton donor and the lipid carbonyl groups. Those lipid C=O groups are, in general, located deeper in the bilayer structure than the phosphate groups (see Figure 2A,C). Other H-bonds can also be formed between the GLUH H donor and O acceptors, corresponding to the glycerol and phosphate lipid head group. H-bond existence maps for all NVT replicas of the GLUH system are provided in the Supporting Information section (Figure S1).

The H-bond between the carboxyl H corresponding to GLU4 and a carbonyl O acceptor of the POPG lipids has a very long lifetime as can be deduced from the H-bond existence maps depicted in Figure S1 of the Supporting Information section. A graphical representation of that H-bond is highlighted in Figure 2C.

It is interesting to note also that in all replicas, the protonated GLU20 residue is able to form a persistent H-bond with a particular lipid phosphate O atom (see Figure S1 and the corresponding H-bond listings). That interaction makes an additional contribution to anchor the protomers to the bilayer interface.

The bilayer structure is distorted by the bound peptides as can be observed in Figure 2, mainly in the upper leaflet, where the peptide is bound. In order to assess the impact of the peptide on the bilayer structure, we have performed several analyses using the SuAVE software tools.^42^

We used a 500 bin resolution that gives a very satisfactory RMSD of 0.07 ± 0.02 nm. The averaged area/lipid calculated with the s_area tool yields similar values for the three systems: GLU (0.70 ± 0.09 nm^2^), GLUH (0.71 ± 0.06 nm^2^), and POPG (0.72 ± 0.06 nm^2^). Other averaged quantities such as bilayer thickness give also very similar values for the three systems. However, the undulations of the bilayer surface attributed to the peptide binding can be detected through the analysis of the local lipid thickness and order using the s_thick and s_order tools, respectively, of the SuAVE software.

**Figure 3:**
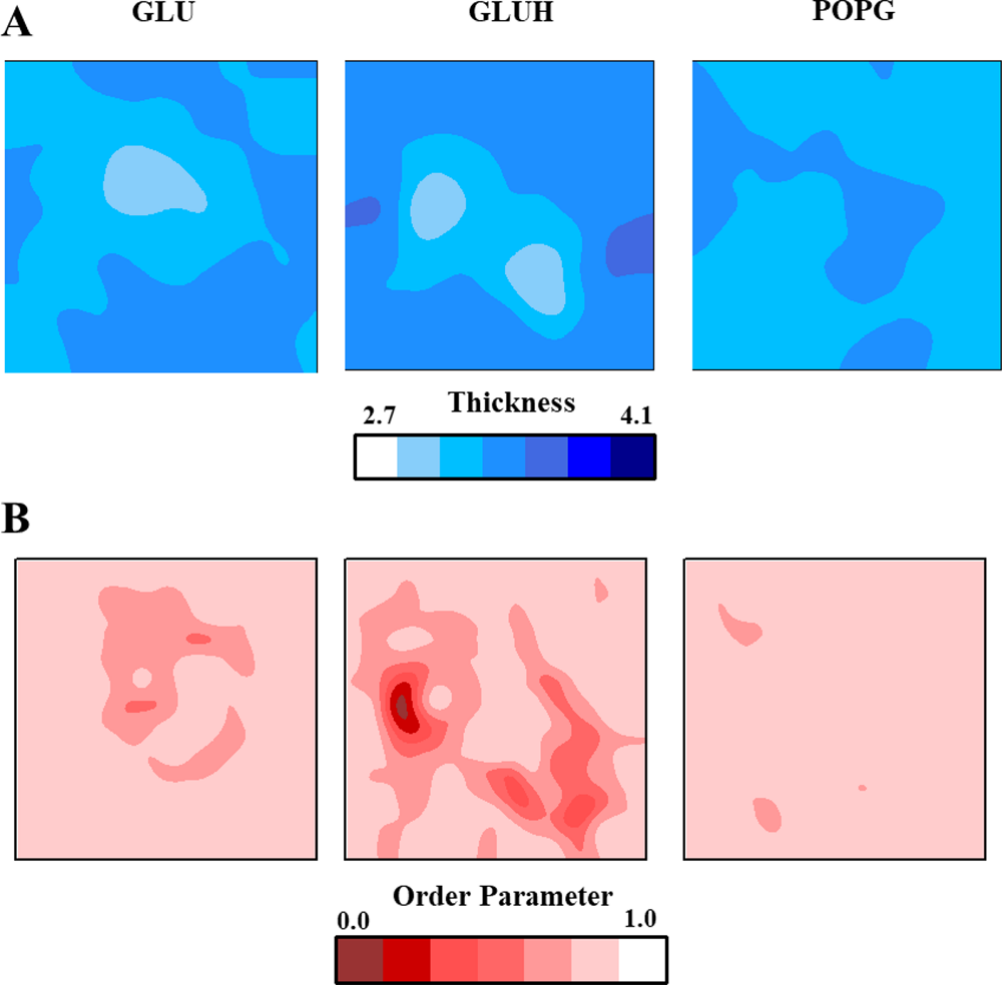
(A) Bidimensional XY map distribution of the bilayer P to P distance calculated with the s_thich SuAVE tool for the GLU, GLUH, and lipid systems, respectively. Values were averaged over the four NVT replicas. (B) Bidimensional XY map distribution of surface order parameters calculated with the s_order SuAVE tool for the GLU, GLUH, and lipid systems, respectively. Values were averaged over the four NVT replicas.

In Figure 3A, P to P distance bidimensional maps evaluated over the grids fitted to the lipid P atoms on each monolayer are depicted for each system. As can be observed, bilayer thickness contractions can be observed under those areas where the peptide binds to the bilayer surface. The effect is more notorious on the GLUH system, where two areas with an average decrease of 0.2 nm with respect to the estimated mean P to P distance of around 3.1 nm can be observed. In the GLU case, one area with a 2.9 nm value can be observed. In addition to this, the SuAVE toolkit allows also the calculation of the order parameter of the fitted surface normal vector with respect to the normal vector of the ideally flat layer surface. A value of 1.0 would correspond to perfectly aligned vectors, while a value of −0.5 would indicate perpendicular ones. The lower the order parameter is the higher the surface curvature. In Figure 3B, bidimensional distributions of order parameters are depicted for the three systems. In parallel with the bilayer thickness data, the peptide-bound systems exhibit areas with enhanced curvature, more notorious in the GLUH case.

**Figure 4:**
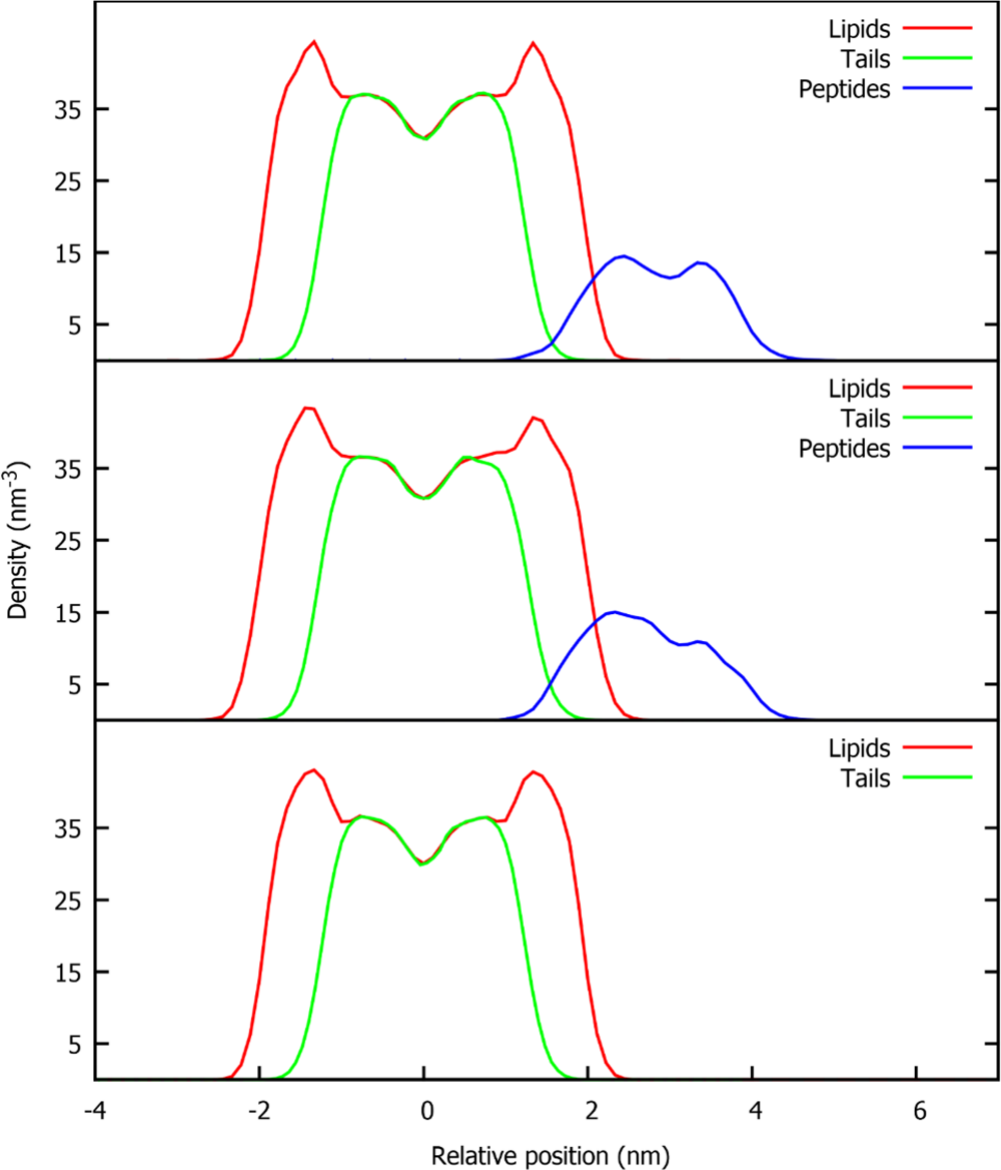
Number density profiles along the bilayer normal. Upper plot: GLU system; central plot: GLUH system; lower plot: pure POPG system.

How those deformations affect the density profiles along the bilayer normal can be assessed by the s_dens tool available in the SuAVE software. It can evaluate the number density profiles corrected taking into account the curvature induced by peptide binding. In Figure 4, number density profiles are shown for the POPG bilayer alone; GLU and GLUH systems were averaged over the four NVT replicas. Minor differences can be observed on the lipid and hydrocarbon tail profiles. Variance can be clearly detected only in the peptide insertion profile, being the protonated peptide (GLUH system) more deeply inserted in the bilayer.

The bilayer deformation caused by the peptide binding is clearly a local phenomenon that can be detected by local evaluation tools. From the information on the P to P distance distribution in Figure 3A, it is possible to estimate the hydrocarbon region thickness. This can be accomplished by subtracting 1.0 nm from the P to P distance as suggested by Huang based on the average P to near hydrocarbon distance (0.5 nm on each layer).^14^ Therefore, an average value of 2.1 nm can be considered for the bulk hydrocarbon region thickness. Taking into account an average decrease of 0.2 nm in those areas where the peptide is bound, the decrease in ratio of Δ*h*/*h* of the hydrocarbon region thickness can be calculated, where *h* is the thickness (2.1 nm) and Δ*h* is the decrement in the bound areas (0.2 nm). The thickness’ decrease in ratio results in −0.1. The hydrocarbon lipid thickness decrement has been used as a measure of the effect caused by the peptide binding and is related to the stretching suffered by the bilayer so that Δ*h*/*h* ≈ −Δ*A*/*A*. The bilayer area increment can be connected to the surface tension through the following relationship:^44^
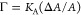
1where Γ is the surface tension, *K*_A_ is the bilayer area compressibility modulus, and Δ*A*/*A* is the relative area increment. For the case of the POPG bilayer, the *K*_A_ modulus was calculated to be 200 ± 20 mN/m, which is in line with the range of values experimentally determined for different lipids.^44,45^ Therefore, the AS-48 binding to the POPG bilayer surface seems to involve the generation of a local surface tension around 20 mN/m. It should be taken into account that the local effect described above may be extended to an additional bilayer surface at higher peptide/lipid ratios not contemplated in this work by reasons given in the Computational Methods section. However, the value is in line with the range (5–15 mN/m) obtained from X-ray data and micropipette aspiration experiments on Giant Unilamellar Vesicles (GUV) by Huang for the cooperative effect of antimicrobial peptide accumulation on DPPC bilayers.^15^

The compact AS-48 structure is reminiscent that of pore-forming peptides (PFP), like BAX, where several amphipathic α-helices pack together in a 3D structure. The effect on the lipid bilayer was proposed to be similar to that exerted by a cluster of single α-helix antimicrobial peptides.^14^ In fact, it has been asserted that the mechanism of action of most antimicrobial peptides by increasing membrane tension is transferable to any membrane active peptide.^46^

**Figure 5:**
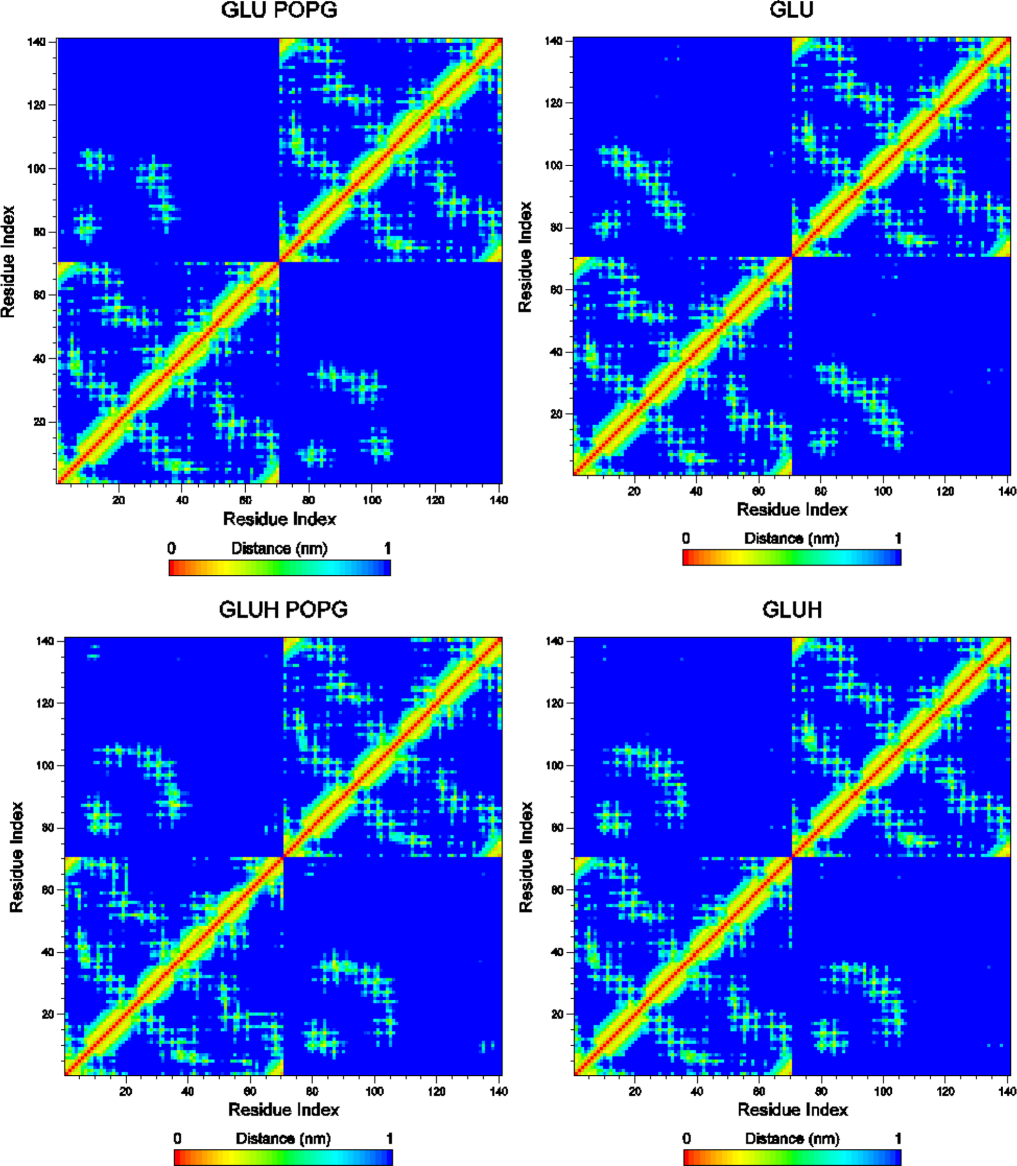
Distance maps between residue groups of the AS-48 dimer. The maps on the right correspond to simulations of the dimer in solution. The maps on the left correspond to averaged maps of the four NVT simulation replicas. The upper and lower plots are for the GLU and GLUH systems, respectively. Residue numbers 1–70 and 71–140 correspond to each protomer, respectively.

Upon binding, the dimer structure of the peptide is slightly modified due to some weakening of the interprotomer interaction between hydrophobic residues. In Figure 5, distance maps between dimer residues are depicted. In each map, the upper left and lower right quadrants correspond to interactions between protomers. Those interactions are circumscribed to hydrophobic residues in the range of 5–40. It can be observed that in the case of the unprotonated peptide bound to the lipid (upper right map in Figure 5), part of those interactions disappears (residues 20–30 of protomer 1 with residues 95–105 corresponding to protomer 2). In the case of the protonated peptide, the interaction loss is less evident.

On the other hand, upon binding, the peptide suffers a notable reduction in mobility. The diffusion coefficient of the AS-48 dimer in water solution was estimated from previous unpublished calculations on a system containing the peptide, water, and counterions. The AS-48 dimer shows a diffusion coefficient of 6.0 ± 1.0 × 10^–7^ cm^2^/s. The peptide diffusion coefficient calculated for the binding step yields a value of 1.0 ± 0.1 × 10^–8^ cm^2^/s for both the protonated and unprotonated peptides. The peptide mobility is reduced about 60 times when bound to the bilayer surface. Diffusion coefficients have been calculated with the gmx msd tool available in Gromacs.

### Poration Mechanism Driven by Surface Tension

The second step of the mechanism studied considers the pore formation mediated by the surface tension exerted by the bound peptide on the membrane and its effective insertion in one bilayer leaflet.

It was already shown that peptide binding to the bilayer surface induced a surface tension on the attacked leaflet increasing, correspondingly, the probability of defect formation.^13,14,16^ In principle, it is expected that the poration process takes place from a micro- to millisecond timescale, far beyond the limits of today’s atomistic or even coarse-grained simulations. One way to boost the poration process within the limits of available computational resources may be to perform simulations with a constant surface tension coupling scheme in the plane of the bilayer. Membrane rupture typically occurs around a few mN/m.^47,48^ The interval of surface tensions associated to antimicrobial binding to membranes is within 5–15 mN/m, as reported by Huang *et al*. based on a theoretical analysis of experimental data.^16^ Consequently, it would be expected that peptide binding to the bilayer surface should produce membrane disruption. However, pore formation preceding membrane rupture is a kinetic process and therefore depends on the loading rate of the applied tension.^47^ Therefore, we performed molecular dynamics simulations at different constant surface tension values on the peptide-bound structures obtained after the equilibration phase produced as a result of the AS-48 binding to the bilayer surface. This strategy has also been used in the study of interactions between lipid bilayers and membrane-stabilizing copolymers.^49^

The aim to perform such simulations in the constant surface tension ensemble was to get successful combinations of temperature–surface-tension at which the poration phenomena could be observed within the limits of the computational models and resources. Initially, we tried different surface tensions at a temperature of 310 K (physiological temperature) for which we only found membrane disruption at surface tension ≥ 70 mN/m. We checked if a 20 K increase would reduce the surface tension value. We obtained that surface tension <60 mN/m did not produce poration at all at 330 K. Therefore, we selected the 310–70 and 330–60 combinations for this study. A more systematic study will be needed to evaluate separately the temperature–surface-tension effects, but this falls out of the scope of the present work.

From now on, the different systems are to be designated by a combination of the system name, the temperature, and the surface tension separated by underscores. Therefore, for example, GLU_310_70 makes reference to the unprotonated GLU system simulated at 310 K temperature and 70 mN/m surface tension.

The constant surface tension applied on the bilayer plane dimensions induces a widening of the membrane according to eq 1, yielding in this case, 35 and 30% area increments for Γ = 70 mN/m and Γ = 60 mN/m, respectively. This widening induces, in turn, an increased probability of membrane defects that can be associated to water molecules that traverse the bilayer.

Lipid separation is mainly produced by two factors, namely, constant surface tension under which simulations are performed and peptide insertion. When the peptide is not inserted, the number of lipids/nm^2^ is 1.3. After peptide insertion, this number decreases to 0.8 and 0.9 lipids/nm^2^ for the systems at surface tensions of 70 and 60 mN/m, respectively. Those calculations were performed with the s_area SuAVE tool. The number of water molecules inside the hydrophobic region of the bilayer was evaluated along each trajectory. The horizontal (in the *xy*-plane) bilayer slice used to count those solvent molecules ranges from *z* = 1.7 to *z* = 2.2 nm, corresponding approximately to the bilayer center. The number of water molecules inside this slice increases from the initial zero value to a constant number after some simulation time depending on the surface tension applied. The approximate time for the initialization of that plateau is around 50 and 25 ns for the Γ = 70 mN/m and Γ = 60 mN/m systems, respectively. In the Supporting Information, a collection of plots representing the time evolution of the number of waters inside the defined slice are included (Figure S2). The end time point for the evaluation of the number of water molecules is just the starting disruption time at which the number of water molecules increases abruptly prior to bilayer collapse (see Table 3 below). Table 2 collects the number of those water molecules averaged over all replicas of each system.

**Table 2:** Average Number of Water Molecules inside the Slice Selected to Represent the Hydrocarbon Lipid Core

system	temperature^a^	surface tension^b^	number of water molecules
GLU	310	70	10 ± 5
	330	60	5 ± 3
GLUH	310	70	10 ± 4
	330	60	4 ± 3
POPG	310	70	1 ± 1
	330	60	1 ± 1

a K.

b mN/m.

The main visual observation of membrane collapse is a sudden large bilayer stretching. Some few nanoseconds earlier, formation of a pore that rapidly grows leading to membrane disruption is visible.

It can be observed that the averaged values are around 10 and 5 water molecules for the 70 and 60 mN/m surface tension simulation systems, respectively (see Table 2). For the pure POPG simulations, bilayer disruption was observed on 3 out of 6 cases, with an almost negligible value of water molecules identified in the bilayer interior in all cases (see the Supporting Information, Figure S2). Lipid bilayer disruption under surface tension is observed even at moderately low tension values (1–25 mN/m).^47^ We show in the present work that the POPG lipid bilayer system without peptides also suffers membrane disruption at the considered surface tensions but less frequently observed in the simulations than the bacteriocin-containing systems. Therefore, the presence of AS-48 induces pore formation that remains metastable in the nanosecond timescale.

**Figure 6:**
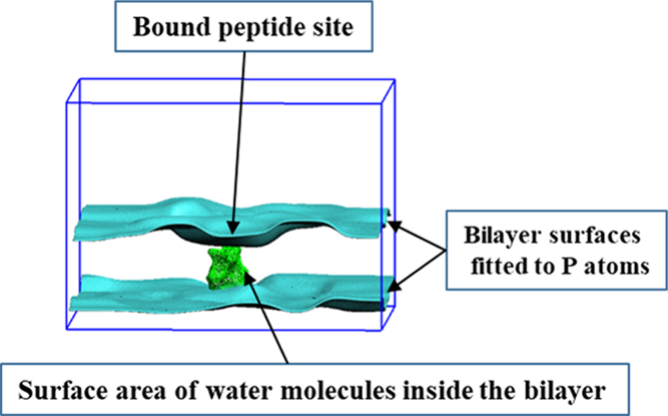
Graphical representation of the water pore formed in the third replica of the GLU_310_70 simulation system. The cyan-colored surfaces were obtained from the SuAVE s_grid tool using 500 points to fit the lipid P atoms. The green-colored surface corresponds to the smoothed VDW surface of water molecules inside the bilayer.

The pore height is defined by a 0.5 nm vertical distance around the bilayer center (1.7 to 2.2 nm in absolute *z*-coordinate values). This region is common to all simulated systems. The water mass density profiles show a uniform distribution in that segment. In order to estimate the pore radius in that region, we assume the bulk water density value (1 g/cm^3^). Figure 6 shows a representation of the water pore. It can be observed that the position of the pore is just below the site where the peptide is bound to the bilayer.

If we assume a cylindrical pore shape of 0.5 nm height and 1 g/cm^3^ water density inside that pore, it is possible to calculate the pore diameter with the following formula:
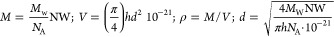
2where *M* is the water mass in gram, *M*_w_ is the water molecular weight, *N*_A_ is Avogadro’s number, NW is the number of water molecules, *V* is the volume in cubic centimeter, *h* is the cylindrical volume height (0.5 nm), and *d* is the cylinder diameter.

This calculation yields diameters of 0.9 ± 0.5 and 0.6 ± 0.3 nm for the 10 and 5 water molecules cases, respectively. An estimated value of 0.7 nm was found experimentally for the pore diameter.^1^ In spite of the approximations taken in the simulations, it can be stated that the simulated result is in line with the experimental one with the following cautions among others. On the one hand, it should be noted that the large fluctuation in the number of water molecules is located in the slice along the different replicas. On the other hand, the pore shape is, someway, an irregular figure, being the vertical slice between 1.7 and 2.2 nm a kind of bottleneck representing a limiting value for the pore size.

The bound peptides trigger, therefore, an increased probability of defect formation in addition to that generated by the applied surface tension, which, in turn, is opportunistically used by the bacteriocin to enlarge such water pores, leading to membrane disruption.

Table 3 summarizes the simulation times at which the bilayer disruption process begins to be noticed. The resulting distribution of time values is concomitant with the stochastic (kinetic) nature of the poration mechanism. Table 3 also collects which protomer faces the lower lipid layer and which charged/polar residues interact with the opposite lipid layer polar head groups allowing pore formation.

**Table 3:** Starting Simulation Time at Which Bilayer Disruption Can Be Observed for the Different Replicas Corresponding to the Essayed Combinations of Temperature and Surface Tension

system	temperature^a^	surface tension^b^	replica	starting disruption time^c^	active protomer	residue
GLU	310	70	1	160	1,2	ARG65,SER37
			2	250	2	ARG48
			3	170	1	LYS64/ARG65
	330	60	1	240	1	ARG65/LYS64
			2	45	1	ARG65
			3	140	1	ARG65
GLUH	310	70	1	160	2	LYS62/LYS61
			2	200	1	SER41
			3	190	2	LYS61/ARG65
	330	60	1	430	1	LYS3
			2	120	1	LYS3
			3	135	2	ARG65
POPG	310	70	1	950	n.a.	n.a.
			2	155	n.a.	n.a.
			3	n.o.	n.a.	n.a.
	330	60	1	n.o.	n.a.	n.a.
			2	170	n.a.	n.a.
			3	n.o.	n.a.	n.a.

a K.

b mN/m.

c ns; n.o.: not observed; n.a.: not applicable.

The kinetic nature of the process is evidenced by the different times at which poration is observed in the replicas (Table 3). One protomer penetrates more deeply, whereas the other tends to stay around the bilayer surface. Only one replica (replica 1, GLU_310_70 system, see Table 3) shows insertion of both protomers into the hydrophobic lipid region but with an asymmetrical movement. One cationic residue of the most penetrating protomer interacts with polar heads of the opposite lipid layer except the polar SER residues in two particular replicas (Table 3).

**Figure 7:**
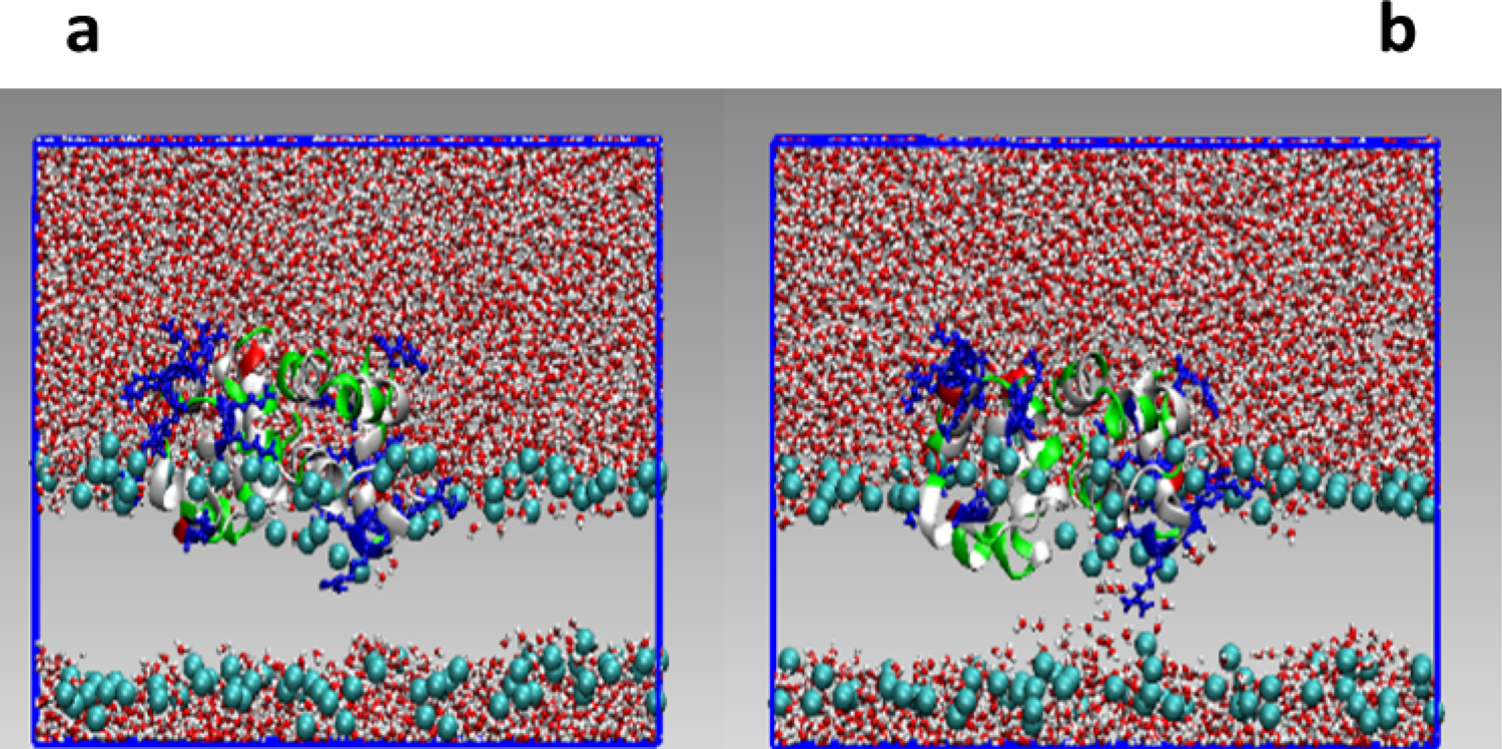
Snapshots at (a) 137 and (b) 170 ns of the third replica corresponding to the GLU_310_70 system. Peptide backbone atoms are shown as ribbons and LYS and ARG side-chain atoms as CPK according to the residue type (red: negatively charged, blue: positively charged, green: polar, and white: hydrophobic). Lipid molecules are not shown for clarity, and phosphorus atoms on the lipids are shown as cyan VDW balls. Water molecules are shown as CPK ball and wires.

The evolution of the poration process exhibited by the different MD replicas is illustrated in Figure 7 with the help of some selected snapshots of a particular simulation. A video of the second replica corresponding to the GLU_330_60 simulation is also included in the Supporting Information (Video 1). The initial binding to the bilayer surface is likely to be driven by electrostatic attraction between the cationic AS-48 residues and the anionic lipid head groups. It can be observed that the AS-48 dimer is able to maintain the contact between both protomers along the binding and bilayer disruption processes. The estimated hydrophobic interaction between protomers should be responsible for the maintenance of the dimeric structure in solution. However, this interaction should be flexible enough to allow the approximate independent movement of one protomer with respect to the other. This observation may be in agreement with the experimental results regarding the importance of those hydrophobic interactions and their flexibility resulting from the bioactivity analysis performed on the AS-48_G13K/L40K_ double mutant.^6^ The hydrophobic residues tend to move toward the apolar lipid bilayer’s interior. Along with this movement, some charged residues are dragged by the hydrophobic residues into the bilayer hydrophobic phase (Figure 7a). Eventually, some of those cationic/polar residues attract the anionic phosphate groups of the lower monolayer, generating a hybrid toroidal pore (Figure 7b). The polar residues interacting with the opposite layer head groups are listed in Table 3.

In the case showed in Figure 7, the interaction between the ARG64 residue and one phosphate lipid group of the lower layer can be observed, starting the formation of a stable pore that subsequently gives rise to membrane disruption.

**Figure 8:**
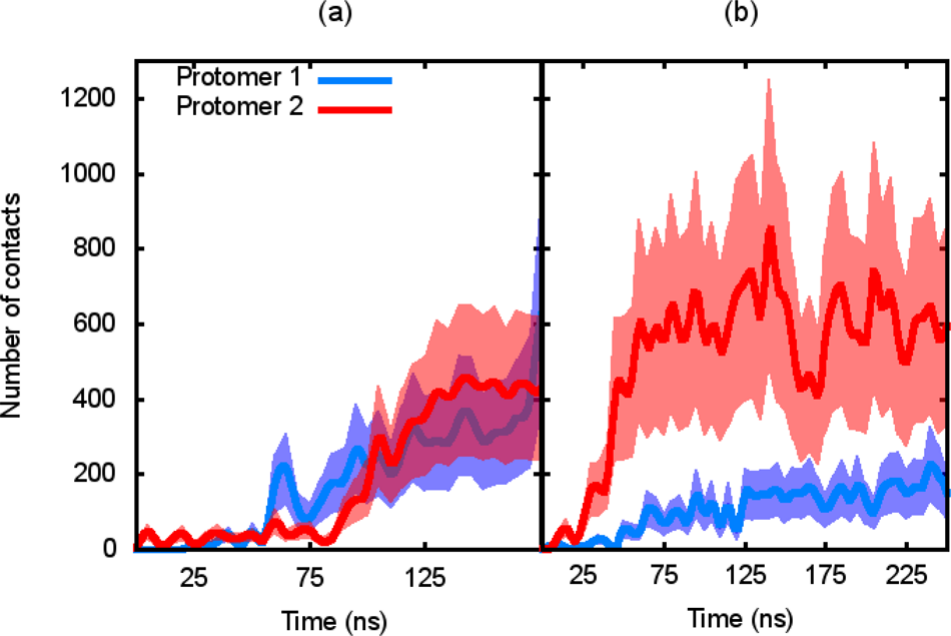
Time evolution of the total number of contacts between AS-48 hydrophobic residues and lipid tails. Each plot corresponds to two replicas of the GLU_310_70 simulated system: (a) replica 1 and (b) replica 2. The solid line represents the mean value, and the shaded areas represent the corresponding standard deviation.

The increase in interactions between the apolar peptide residues and the lipid tails can be illustrated by the time evolution of the total number of contacts between those groups. Figure 8 shows the number of contacts evaluated when the distance between atoms of those groups is within 6 Å.

In general, the number of contacts between the AS-48 hydrophobic residues and the lipid tails that grows along the dynamics simulation is in agreement with the expected propensity of the hydrophobic residues to interact with the apolar bilayer interior. Two different tendencies can be observed in the corresponding plots depicted in Figure 8 and in the Supporting Information (Figure S3). In the first one, the two protomers increase gradually the number of contacts almost in parallel (replicas 1 and 3 for the GLU_310_70 system, replica 3 for the GLU_330_60 system, replicas 1 and 3 for the GLUH_310_70 system, and replica 3 for the GLUH_330_60 system, see Figure 8a and the Supporting Information, Figure S3). In the other, one protomer presents an abrupt increase in the number of contacts larger than the other, which is approximately maintained till the bilayer collapse (replica 2 for the GLU_310_70 system, replicas 2 and 3 for the GLU_330_60 system, replica 2 for the GLUH_310_70 system, and replicas 1 and 2 for the GLUH_330_60 system, see Figure 8b and the Supporting Information, Figure S3).

Comparing the information in Table 3, Figure 8, and corresponding figure in the Supporting Information (Figure S3), it can be observed that in the two cases, the protomer with a larger number of hydrophobic contacts does not clearly correspond to the protomer containing the polar residue interacting with the lower bilayer head groups (replicas 2 and 3 of the GLU_330_60 system). The progressive insertion of the hydrophobic moiety into the lipid bilayer may be a necessary but not sufficient condition to yield membrane poration.

The polar residues responsible for the interaction with the lower lipid layer head group, which drives pore formation, are, in general, cationic LYS or ARG amino acids located in helices 4 and 5. Either one or two of such residues are sufficient to promote distortion of the lower lipid layer by attracting phosphate groups to the bilayer center. Only two replicas show a polar SER residue involved in the formation of hydrogen bonds with O acceptors in the phosphate groups located in the lower lipid layer (replica 1 of the GLU_310_70 and replica 2 of the GLUH_310_70 systems, see Table 3).

The time evolution of the normal to the bilayer position of the center of mass corresponding to each cationic residue can be found in the Supporting Information section, Figure S4. All graphs show the bilayer thinning produced during the initial 20–30 ns by the applied surface tension. The *z* position of the center of mass (COM) corresponding to the P atoms in the upper monolayer depicts a parabolic decrease until a plateau value is maintained, whereas the lower P atom layer presents an almost stable average value (see Figure S4). It can be observed, in general, that one or two cationic residues are able to reach interaction with the anionic head groups of the lower lipid layer, as was previously explained. The remaining cationic amino acids stay in the upper lipid/water interface. Most of the replicas also show one protomer inserting more deeply into the bilayer interior. Particularly interesting is the case corresponding to the first replica of the GLU_310_70 system. In that plot, it can be observed that residue ARG65 of protomer 1 is below the average lipid P atom positions from almost the beginning. It continues a slow progression toward the center of the bilayer until its interaction with the lower phosphate layer groups, near the membrane collapse point. On the other hand, residue ARG48 of protomer 2 remains in the aqueous phase above the P atom layer until it descends toward the bilayer interior in the last 50 ns. Actually, SER37 is the identified residue interacting with the lower lipid monolayer, but ARG48 movement serves to illustrate the other observed behavior of cationic residues that suddenly pass through the bilayer interior from the water phase. This behavior can be observed also in replica 2 of the same system (see the Supporting Information section, Figure S4). Therefore, it can be remarked that one of the protomers is more involved in the poration process itself. However, the other one is not only a mere spectator peptide for two reasons. On the one hand, it contributes to membrane defect formation by incorporating hydrophobic interactions between its hydrophobic residues and the lipid tails. On the other hand, its charged and polar residues add extra stabilization to the poration process by their electrostatic and H-bridging interactions with the water phase.

We have evaluated the number of H-bonds between the two protomers and the water solvent (Figure S5 in the Supporting Information shows the results for all systems). It can be observed that the spectator protomer presents, in general, a higher number of H-bonds with the solvent compared to the other protomer during the simulation time prior to pore expansion and membrane disruption. Those numbers are smaller (in ranges of 70–95 and 60–85 for the GLU and GLUH systems, respectively; see Figure S5) than the average number of H-bonds per frame of the DF-I dimer in water solution, 103 ± 5, calculated from unpublished MD simulations.

Another interesting observation regarding the poration process in the unprotonated GLU systems concerns the interaction between the TRP24 amino acids of both protomers. Experimental data show that the presence of TRP24 greatly contributes to the antimicrobial activity of AS-48, whereas the second tryptophan (TRP70) seems more related to the post-translational processing rather than having a direct role in the killing mechanism.^5^ Those TRP24 residues are located between hydrophobic helices 1 and 2 in a highly conserved hydrophobic region among circular bacteriocins. The crystallographic structures corresponding to the DF-I dimeric form (PDB-ID: 1O82) show the TRP24 residues in close proximity. After the first binding phase, both amino acids are far apart due to the relative rotation of both protomers to favor the electrostatic interaction between the peptides and the bilayer surface. During the surface-tension simulations, another rotation between both protomers approaches both TRP24 residues. This can be observed in Figure S6 where the distance between the COM of both TRP24 residues is plotted along the simulation time. At the same time, that amino acid pair is progressively displaced to the top bilayer surface plane until membrane disruption occurs. In Figure S6, the time evolutions of the COM *z*-coordinate corresponding to each TRP residue in both protomers are shown for different replicas corresponding to the unprotonated system. However, as can be observed, the two TRP70 amino acids of both protomers remain around the lipid bilayer surface marked by the P atom *z*-positions (see Video 2 in the Supporting Information). Those residues belong to helix 5, in the vicinity of cationic amino acids that are involved in accessing the membrane–water interface, being responsible for the union to the host cells by interactions with the anionic lipid head groups. The distance between those two TRP70 residues stays larger than 2 nm along any of the simulations. The position of the four TRPs lies approximately in the same plane parallel to the bilayer surface. In that spatial configuration, cationic ARG and LYS residues of one protomer can interact with the lipid head groups of the lower leaflet, initiating pore formation. This is the case in 5 out of 6 simulations, as can be observed in Table 3. Video 2 in the Supporting Information illustrates the TRP24–TRP24 approximation along one selected simulation. The close proximity between the TRP24 residues cannot be observed in any of the GLUH simulations due to the anchor effect of the H-bonds involving the protonated GLU20 amino acids, restricting the relative orientation of both protomers.

The TRP–TRP interaction has been commonly identified as a stabilizing contribution in diverse bio-structural events like protein folding and self-assembly.^50^ Several experimental pieces of evidence remark the important role of tryptophan in the antimicrobial activity of different peptides^51^ and bacteriocins.^52^ A particular feature of the interaction with bilayers is the preferred position of TRP residues near the lipid bilayer surface, as occurred with TRP24 in this case.

In summary, the TRP24–TRP24 interaction helps to promote pore formation by forcing the protomers to enter deeper into the lipid bilayer and orienting one protomer so that some of its cationic residues on helices 4 and 5 interact with the opposite layer polar lipid head groups.

## Conclusions

All-atomistic molecular dynamics simulations have been used to gain insights into the molecular mechanism of action of the AS-48 bacteriocin in a bacterial membrane model. An anionic lipid bilayer was selected to represent the bacterial membrane, as it is a distinctive feature of those microorganisms with respect to eukaryotic cell membranes.

A two-step mechanism has been explored to explain the AS-48 bioactivity, namely, peptide binding to the membrane bilayer and surface-tension-mediated poration. During the first stage, the AS-48 peptide binds readily to the bilayer surface thanks to the electrostatic attraction between the cationic residues and the anionic lipid polar head groups. LYS and ARG charged amino acids are clustered in helices 4 and 5 of the bacteriocin, yielding a local charge density comparable to other antimicrobial peptides. In addition, the AS-48 bacteriocin contains four GLU residues that show an important role when protonated at low pH values. Those residues are able to form H-bonds with different lipid polar groups, enhancing the distorting effect of the antimicrobial peptide on the bilayer structure. The lipid hydrocarbon core suffers a thinning process compatible with an increase of the bilayer surface tension, which may be ultimately responsible for membrane disruption.

In the second stage, the poration process induced by increased surface tension was also explored. This is based on the fact that peptide binding to the bilayer surface generates surface tension in a range that causes membrane poration, as deduced from several experimental observations. Atomistic simulation at constant surface tension was performed in a system with a peptide-to-lipid ratio of 1/64. The variable simulation time at which membrane poration takes place indicated that this is a stochastic (kinetic) process. The mechanism observed in the simulations starts from the peptide bound to the lipid bilayer surface state. As the bilayer surface stretches due to the applied surface tension, some defects are generated on the bilayer lamellar structure. The AS-48 hydrophobic residues penetrate the bilayer to accommodate the lipid hydrocarbon interior. Some charged or polar residues are dragged to interact with the polar head groups of the lower lipid layer, generating a water pore and giving rise to membrane disruption. In general, one protomer directly participates in pore formation. The other protomer supports its action by adding hydrophobic interactions to increase membrane defects and helps to stabilize the interaction with the water phase. The protonation state of the GLU residues seems to have no effect in this stage of the poration process.

In this work, it has been shown that surface tension, generated by the accumulation of peptides on the bilayer surface, is essential in the mechanism of action of antimicrobial peptides such as the AS-48 bacteriocin. The different residue types play a particular role in the antimicrobial mechanism. The cationic amino acids are responsible for the favorable binding to the anionic bilayer surface, on the one hand, and actively contribute to pore formation by electrostatically attracting polar head groups of the lower leaflet to the bilayer interior. The GLU residues, when protonated, enhance the distorting effect on the upper lipid layer by the formation of H-bonds with lipid carbonyl groups. Finally, hydrophobic amino acids are able to penetrate the bilayer interior to favorably interact with the hydrocarbon lipid core, dragging those cationic residues to build metastable pores that ultimately lead to membrane disruption. In particular, it has been shown that TRP24 residues in the unprotonated GLU systems play an important role in the poration mechanism due to the TRP24–TRP24 interaction that contributes to the orientation of the peptide to interact with the lipid opposite layer. This result gives a plausible explanation for the activity drop experimentally observed in TRP24ALA mutations.

The experimental data obtained with AS-48 double mutations suggest that dimer dissociation (DF-II form) should be a necessary step for bioactivity. We have not observed the complete dissociation of the AS-48 dimer within the timescale of the simulations. However, the simulations clearly show that the hydrophobic–hydrophobic contacts between residues, characteristic of the native DF-I structure, are weakened to accomplish the poration process.

Taking into account all this information, some structural modifications may be proposed to enhance the antibacterial bioactivity of such bacteriocins: (1) Increase the number of cationic residues on helix 4 and especially on helix 5. Charge clustering has been experimentally shown to improve antimicrobial activity in simple α-helical peptides. (2) Increase also the number of GLU amino acids in the boundaries of the cationic clusters to enhance the bilayer defect formation. Other amino acids with H-bond donor capacity may be also interesting in those positions.

## Supporting Information Available

(Figure S1) H-bond existence maps for the interaction between GLU residues and lipid polar headgroups are presented; (Figure S2) the number of water molecules in the hydrophobic central slice defined by the normal to the bilayer *z*-coordinate (1.7 nm < *z* < 2.2 nm); (Figure S3) time evolution of the number of contacts between AS-48 hydrophobic residues and lipid tails, individual plots for each replica; (Figure S4) plots describing the time evolution of the normal to the bilayer position of the center of mass corresponding to each cationic residue; (Figure S5) plots describing the time evolution of the number of H-bonds between each AS-48 protomer and the water molecules; (Figure S6) plots describing the time evolution of the COM corresponding to TRP24 residues along with the distance between those amino acids (PDF)
(Video 1) Second replica of the GLU_330_60 simulation; protein is represented by the backbone with colors according to the residue type (red: negatively charged, blue: positively charged, green: polar, and white: hydrophobic); cationic residues also included their side chain; P lipid atoms are represented as cyan VDW spheres; Water O atoms as pink-colored points (MPG)
(Video 2) Second replica of the GLU_330_60 simulation; protein is represented by the backbone with colors according to the residue type (red: negatively charged. blue: positively charged, green: polar, and white: hydrophobic); TRP24 and TRP70 residues are represented in yellow and orange VDW spheres, respectively; P lipid atoms are represented as cyan VDW spheres; water molecules are not represented (MPG)

## Supplementary Material

ci1c00838_si_001.pdf

ci1c00838_si_002.mpg

ci1c00838_si_003.mpg

## Notes

The software used in this work is available in the public domain: Gromacs2016, SuAVE 1.0, VMD 1.9.3, and Gnuplot 4.6. Structures (.gro format) and MD simulation trajectories (.xtc format) of all systems described in the manuscript are available upon reasonable request to authors. Also available are topology files (.top and .itp formats) and Gromacs run parameters files (*.mdp).
