# Supplementary material for: The Role of Key Amino Acids in the Antimicrobial Mechanism of a Bacteriocin Model Revealed by Molecular Simulations
Source: J Chem Inf Model. 2021 Dec 7;61(12):6066–78. doi: 10.1021/acs.jcim.1c00838 (PMC9178794; doi:10.1021/acs.jcim.1c00838)

The Role of Key Amino Acids in the Antimicrobial Mechanism of a Bacteriocin Model Revealed by Molecular Simulations

Víctor L. Cruz[1,*], Javier Ramos[1] , Javier Martinez-Salazar[1], Manuel Montalban-Lopez[2], Mercedes Maqueda[2]

[1] BIOPHYM, Department of Macromolecular Physics, Instituto de Estructura de la Materia, IEM-CSIC, C/ Serrano 113 bis, 28006 Madrid, Spain

[2] Department of Microbiology, University of Granada, C/ Fuentenueva s/n, 18071 Granada, Spain

* Correspondence: vl.cruz@csic.es

Supporting Information

Figure S1. Hydrogen Bond Existence Maps

H-bond between GLU residues and lipid O atoms in the GLUH system are represented as existence maps along the NVT simulation time for the different replicas.

The listing below each map tabulates the index number used in the plot in terms of the donor GLU residue, where the H atom in the carboxyl GLU sidechain acts as donor atom and acceptor lipid molecule with specificity of each atom type. The GLU residue numbers in the upper part of the table below to protomer one.

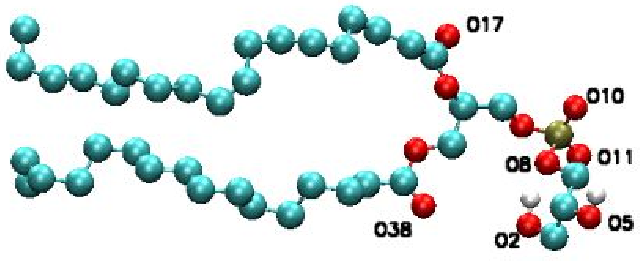

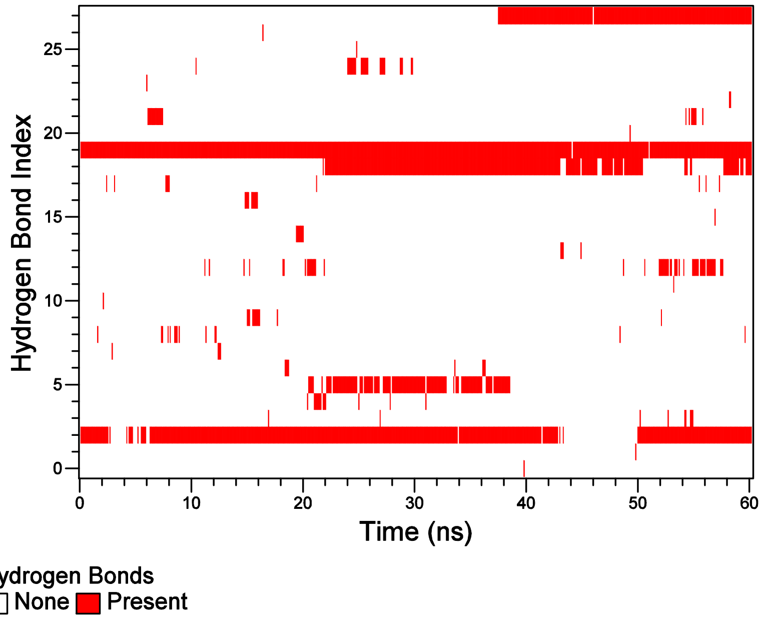

Index	Donor Residue	Acceptor Residue	Acceptor Atom
0	4GLU	157LPOPG	O2
1	4GLU	171LPOPG	O2
2	4GLU	171LPOPG	O17
3	58GLU	89DPOPG	O2
4	58GLU	89DPOPG	O8
5	58GLU	89DPOPG	O10
6	58GLU	89DPOPG	O11
7	58GLU	171LPOPG	O2
8	58GLU	171LPOPG	O5
9	58GLU	172LPOPG	O2
10	58GLU	173LPOPG	O11
11	4GLU	105DPOPG	O8
12	4GLU	105DPOPG	O11
13	4GLU	107DPOPG	O5
14	4GLU	107DPOPG	O11
15	4GLU	146LPOPG	O2
16	4GLU	146LPOPG	O5
17	4GLU	150LPOPG	O2
18	4GLU	150LPOPG	O11
19	20GLU	174LPOPG	O10
20	49GLU	148LPOPG	O5
21	49GLU	148LPOPG	O11
22	49GLU	148LPOPG	O38
23	58GLU	146LPOPG	O2
24	58GLU	154LPOPG	O2
25	58GLU	154LPOPG	O5
26	58GLU	154LPOPG	O10
27	58GLU	154LPOPG	O11

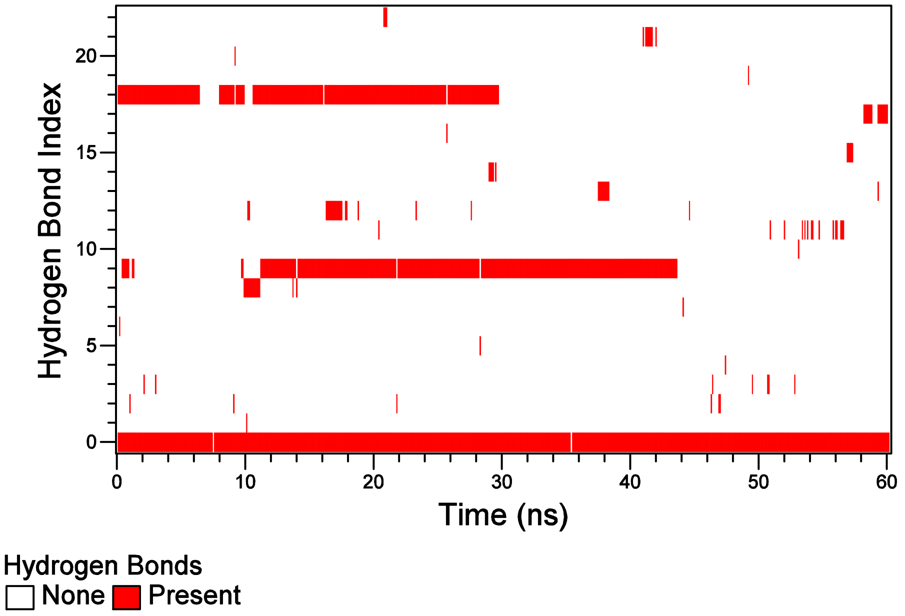

Index	Donor Residue	Acceptor Residue	Acceptor Atom
0	4GLU	171LPOPG	O17
1	49GLU	90DPOPG	O5
2	58GLU	171LPOPG	O2
3	58GLU	171LPOPG	O5
4	58GLU	171LPOPG	O10
5	58GLU	172LPOPG	O5
6	58GLU	173LPOPG	O2
7	58GLU	173LPOPG	O5
8	58GLU	173LPOPG	O8
9	58GLU	173LPOPG	O11
10	4GLU	105DPOPG	O8
11	4GLU	105DPOPG	O11
12	4GLU	150LPOPG	O2
13	4GLU	150LPOPG	O5
14	4GLU	150LPOPG	O8
15	4GLU	150LPOPG	O10
16	20GLU	98DPOPG	O2
17	20GLU	126DPOPG	O2
18	20GLU	174LPOPG	O10
19	49GLU	84DPOPG	O11
20	49GLU	127DPOPG	O5
21	58GLU	84DPOPG	O10
22	58GLU	154LPOPG	O2

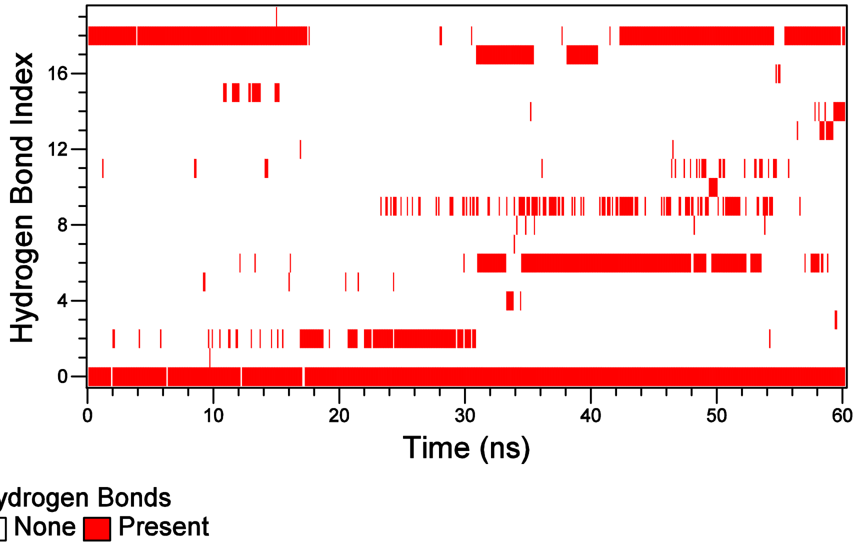

Index	Donor Residue	Acceptor Residue	Acceptor Atom
0	4GLU	171LPOPG	O17
1	58GLU	90DPOPG	O5
2	58GLU	171LPOPG	O5
3	58GLU	173LPOPG	O5
4	58GLU	173LPOPG	O8
5	58GLU	173LPOPG	O10
6	58GLU	173LPOPG	O11
7	58GLU	173LPOPG	O12
8	4GLU	105DPOPG	O8
9	4GLU	105DPOPG	O11
10	4GLU	120DPOPG	O11
11	4GLU	150LPOPG	O2
12	4GLU	150LPOPG	O5
13	4GLU	150LPOPG	O8
14	4GLU	150LPOPG	O10
15	4GLU	150LPOPG	O11
16	20GLU	98DPOPG	O5
17	20GLU	128DPOPG	O17
18	20GLU	174LPOPG	O10
19	58GLU	154LPOPG	O2

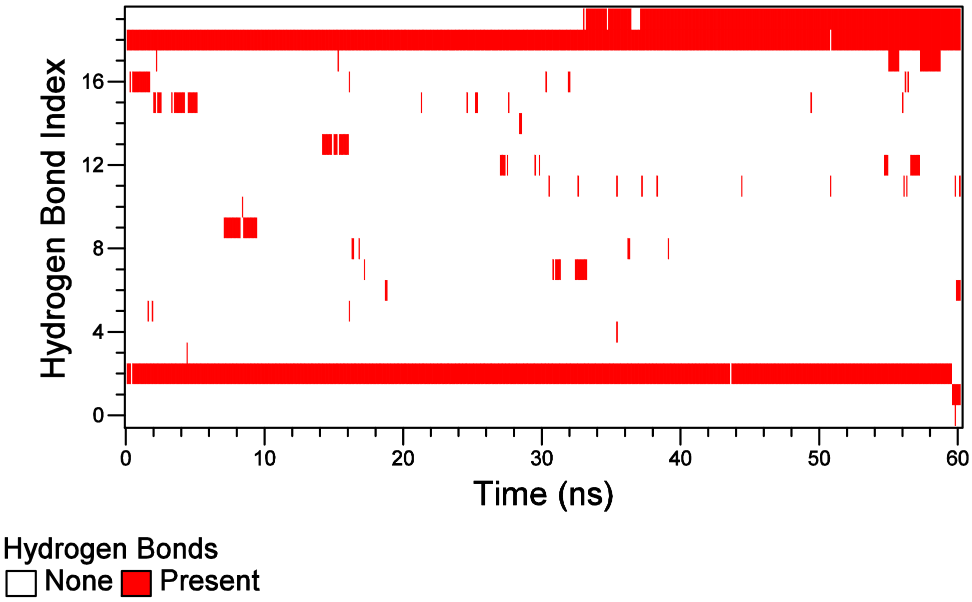

Index	Donor Residue	Acceptor Residue	Acceptor Atom
0	4GLU	171LPOPG	O8
1	4GLU	171LPOPG	O11
2	4GLU	171LPOPG	O17
3	49GLU	173LPOPG	O2
4	58GLU	88DPOPG	O2
5	58GLU	171LPOPG	O5
6	58GLU	171LPOPG	O10
7	58GLU	172LPOPG	O2
8	58GLU	173LPOPG	O2
9	58GLU	173LPOPG	O10
10	58GLU	173LPOPG	O11
11	4GLU	105DPOPG	O11
12	4GLU	107DPOPG	O2
13	4GLU	107DPOPG	O5
14	4GLU	107DPOPG	O10
15	4GLU	150LPOPG	O2
16	4GLU	150LPOPG	O5
17	4GLU	150LPOPG	O11
18	20GLU	174LPOPG	O10
19	58GLU	154LPOPG	O11

Figure S2. Number of water molecules in the hydrophobic central slice defined by the normal to the bilayer z-coordinate (1.7 nm < z < 2.2 nm). (a) replicas for the GLU_310_70 system. (b) Idem for the GLU_330_60 system. (c) Idem for the GLUH_310_70 system. (d) Idem for the GLUH_330_60 system. (e) Idem for the POPG_310_70 system. (f) Idem for the POPG_330_60 system.

(a)

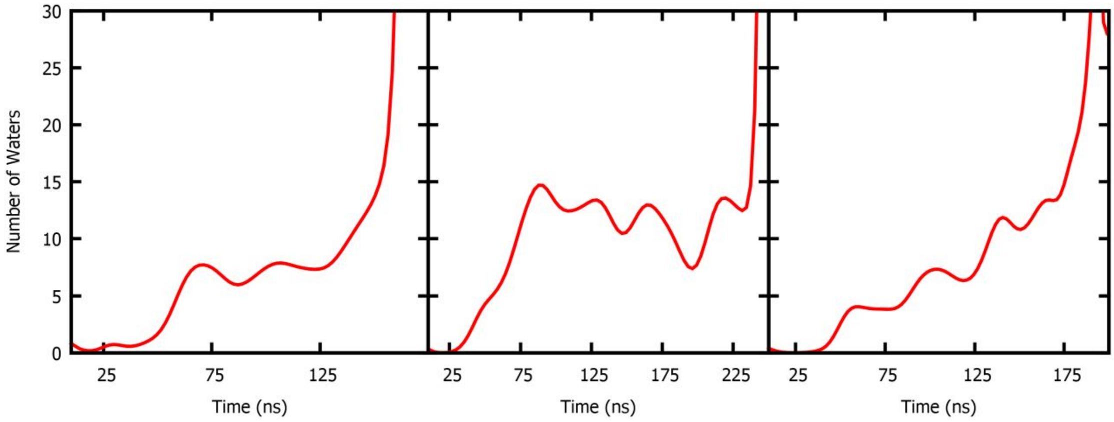

(b)

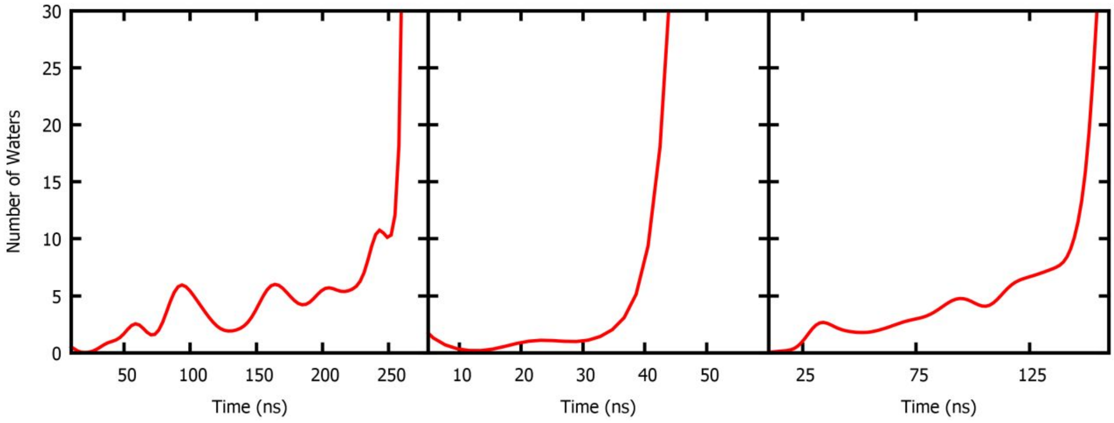

(c)

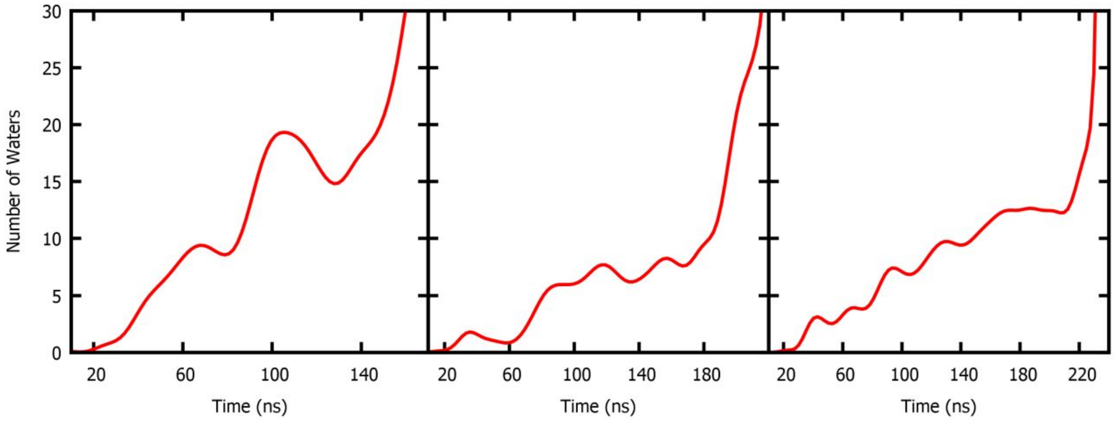

(d)

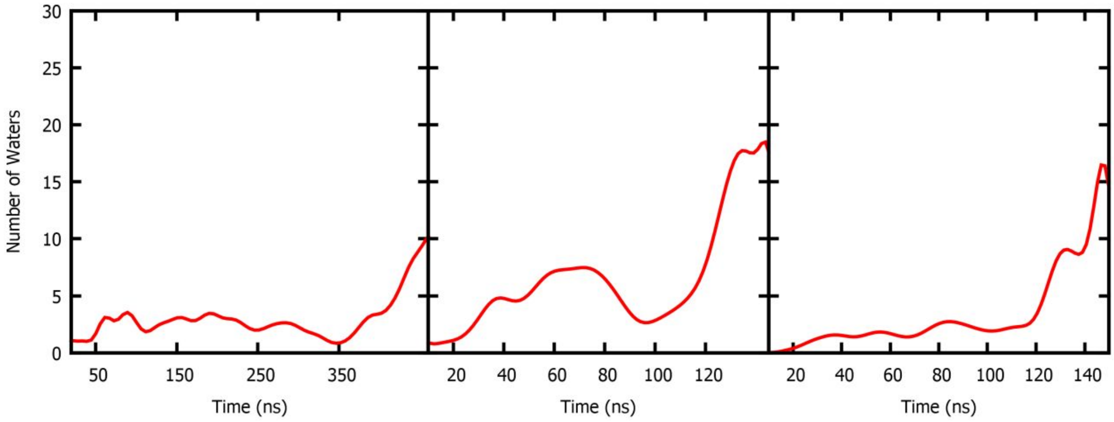
Number of Waters
0
5
10
15
20
25
30
50
150
250
350
Time (ns)
20
40
60
80
100
120
Time (ns)
20
40
60
80
100
120
140
Time (ns)

(e)

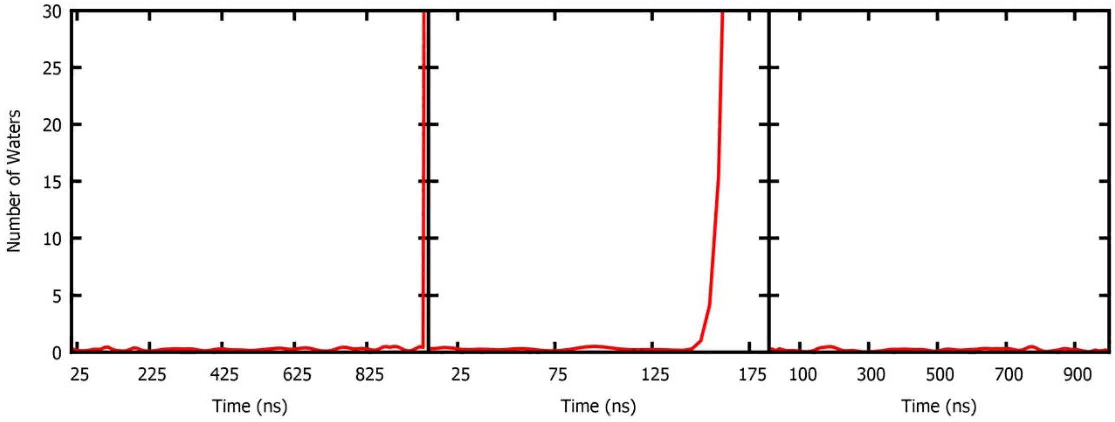
Number of Waters
0
5
10
15
20
25
30
25
225
425
625
825
Time (ns)
25
75
125
175
Time (ns)
100
300
500
700
900
Time (ns)

(f)

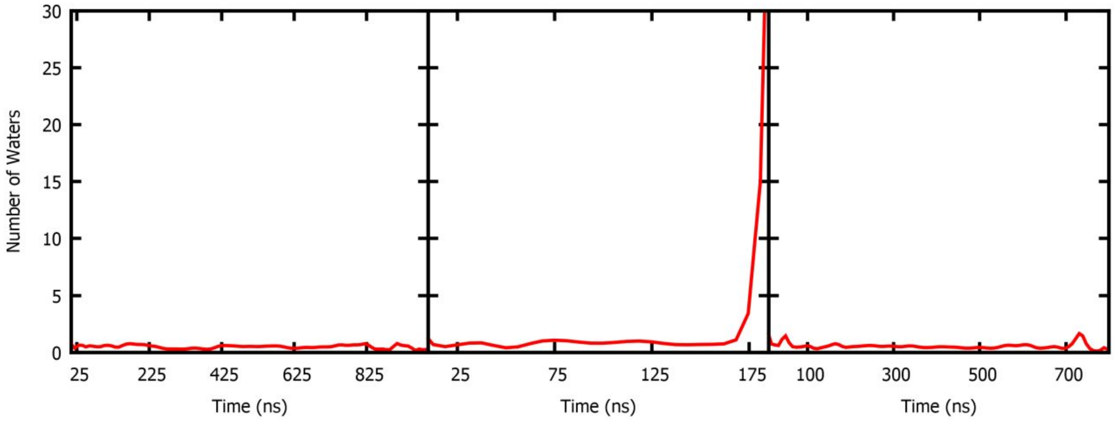
Number of Waters
0
5
10
15
20
25
30
25
225
425
625
825
Time (ns)
25
75
125
175
Time (ns)
100
300
500
700
Time (ns)

Figure S3. Time evolution of the number of contacts between AS-48 hydrophobic residues and lipid tails. Each row corresponds to the three replicas of the different simulated systems: (a) GLU_310_70; (b) GLU_330_60; (c) GLUH_310_70 and (d) GLUH_330_60. Those Figures correspond to Figure 6 in the main text. All cases are reproduced here for completeness with the raw data.

(a)

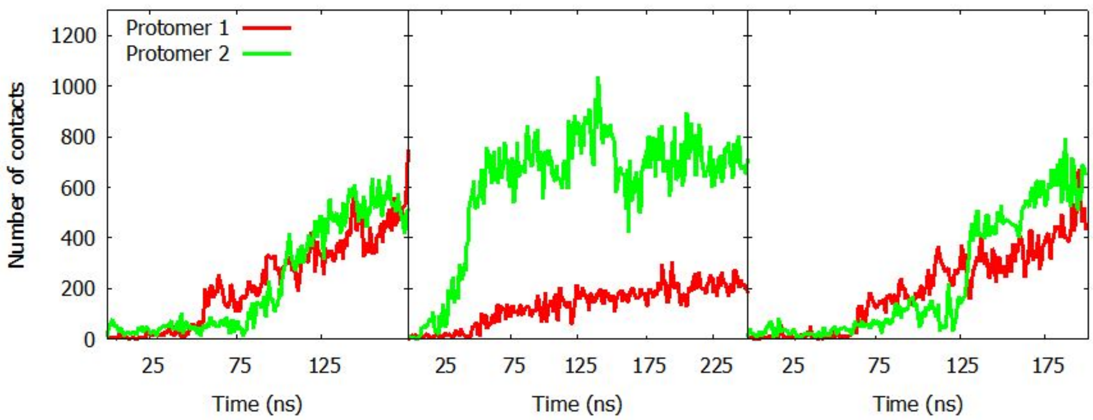

(b)

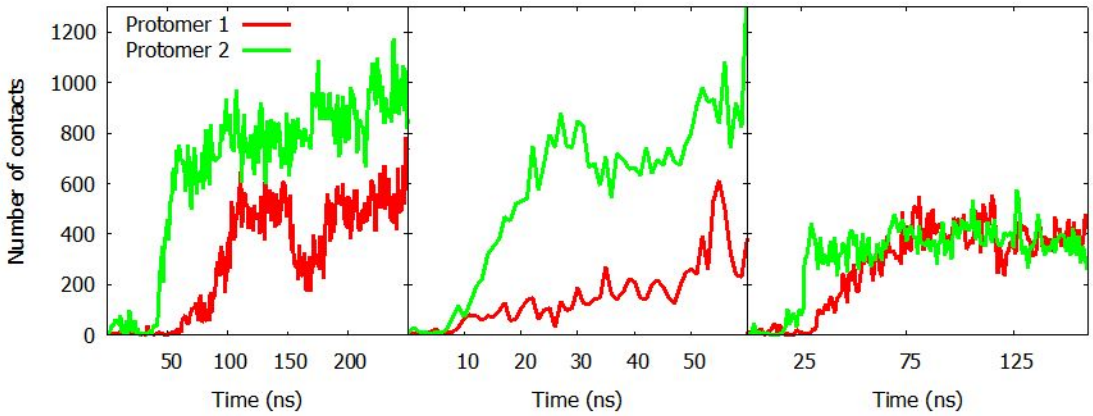

(c)

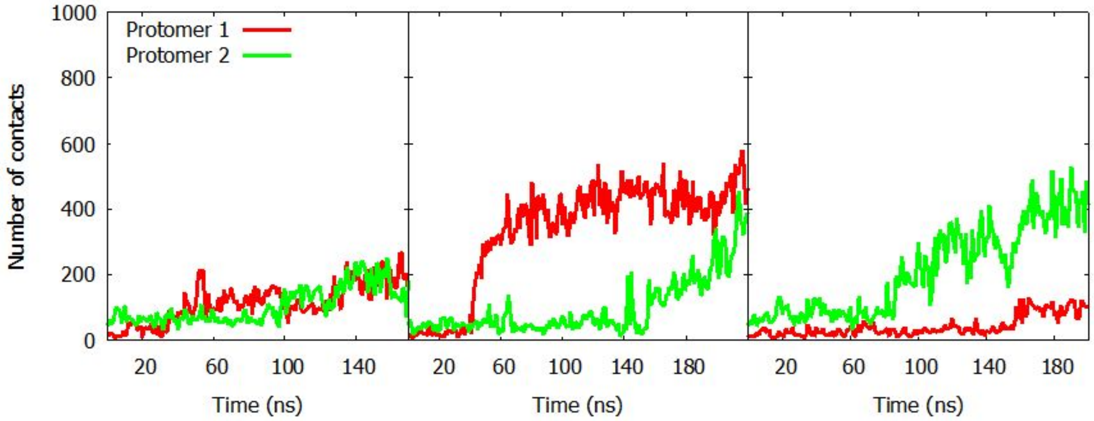

(d)

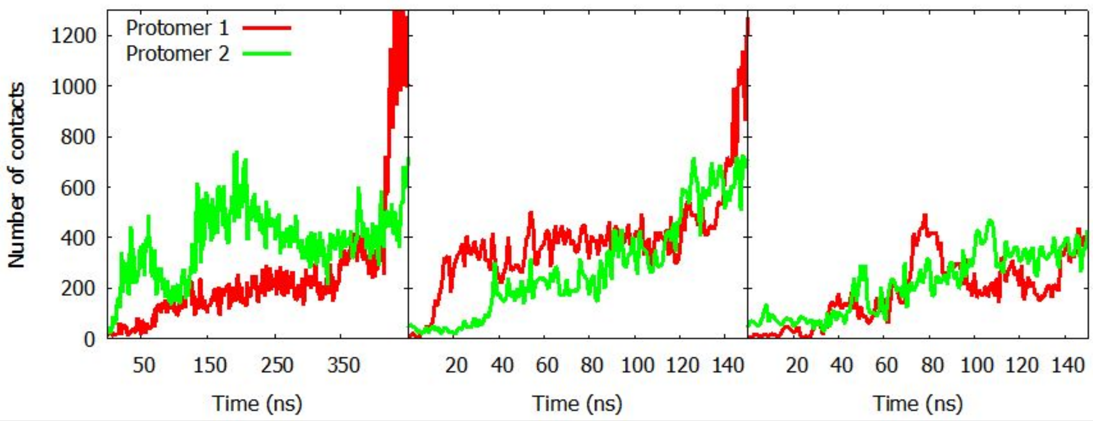

Protomer 1
Protomer 2
Number of contacts
0
200
400
600
800
1000
1200
50
150
250
350
Time (ns)
20
40
60
80
100
120
140
Time (ns)
20
40
60
80
100
120
140
Time (ns)

Figure S4. Time evolution of the z-coordinate corresponding to cationic residues COM. Left and right graphs correspond to protomer 1 and protomer 2 respectively. The transparent gray area corresponds to the distance between P atoms of both bilayer leaflets.

GLU_310_70. Replica 1

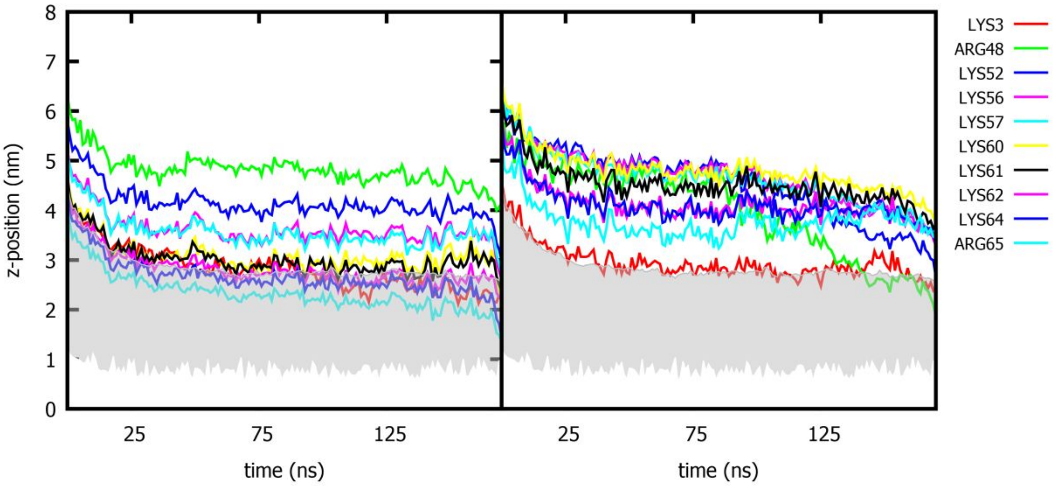

GLU_310_70. Replica 2

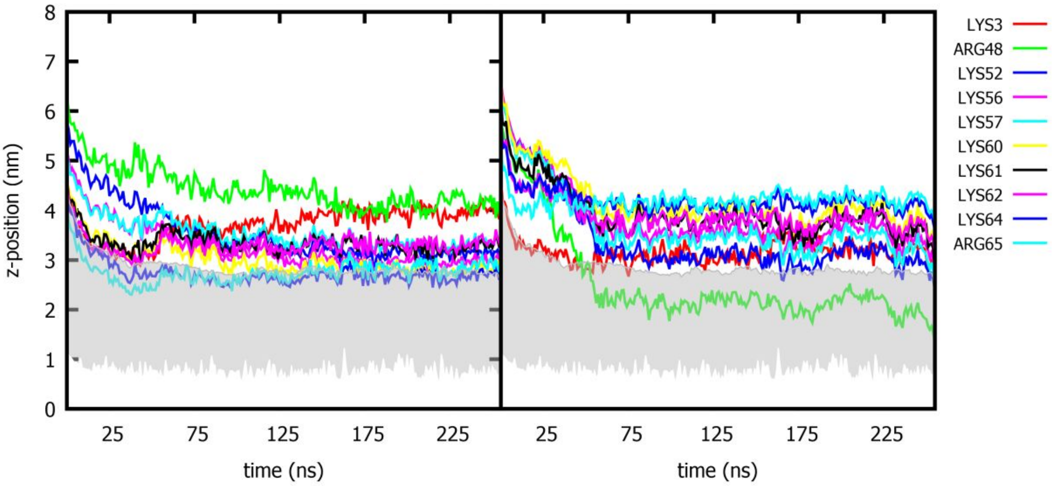

GLU_310_70. Replica 3

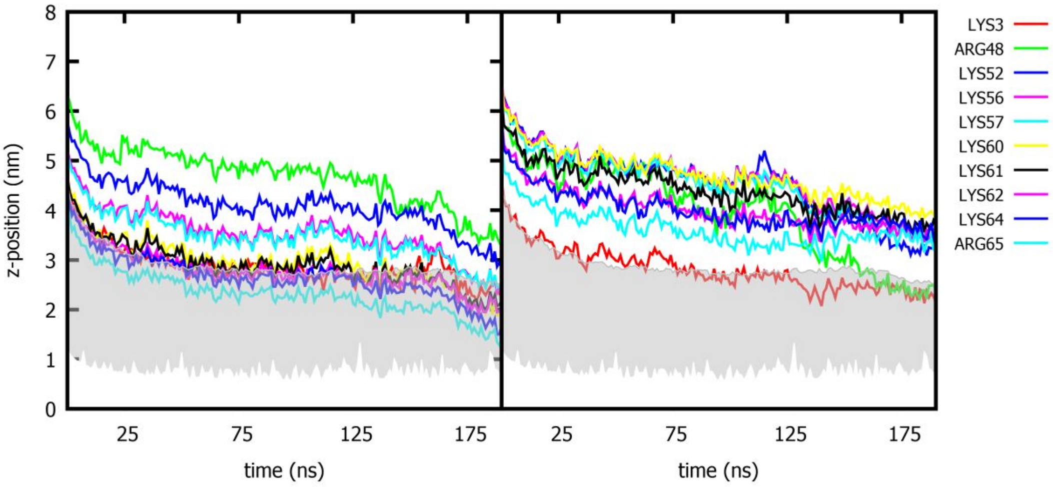

GLU_330_60. Replica 1

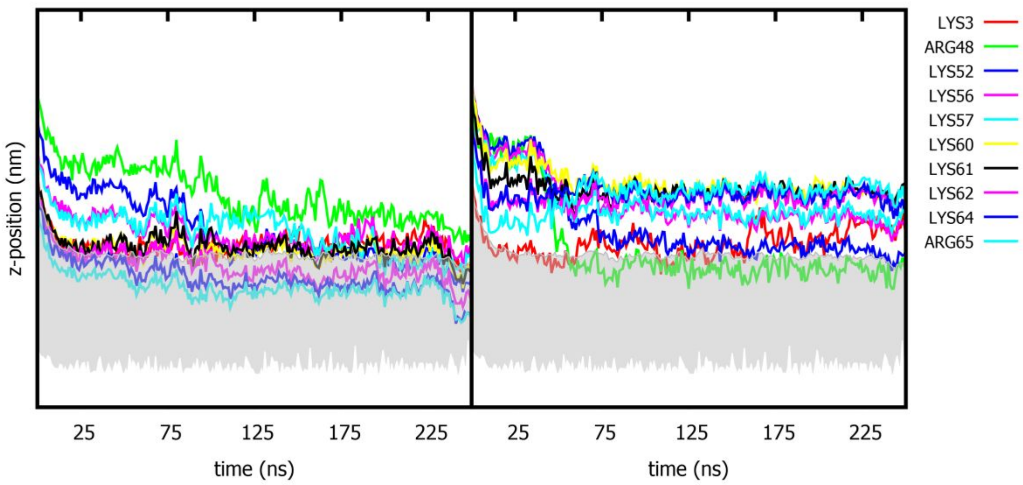

GLU_330_60. Replica 2

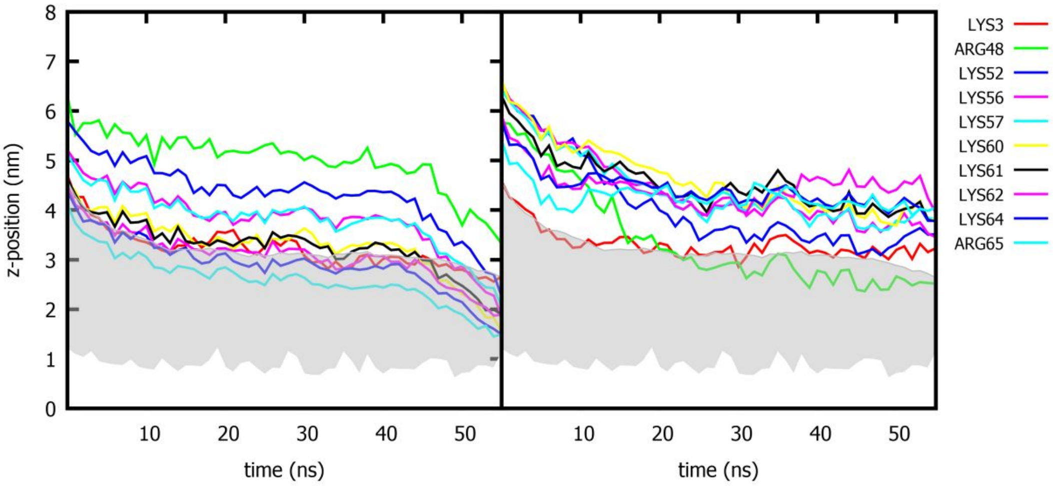

GLU_330_60. Replica 3

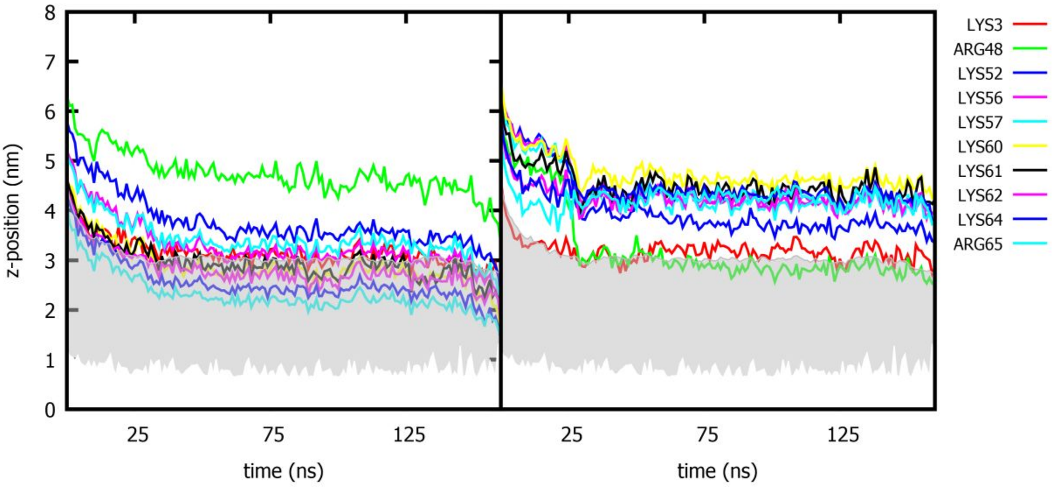

GLUH_310_70. Replica 1

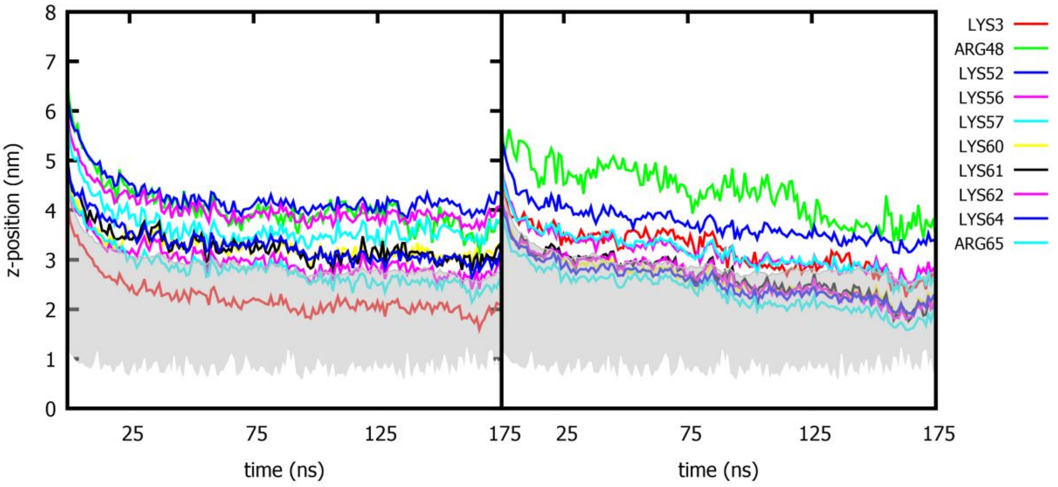

GLUH_310_70. Replica 2

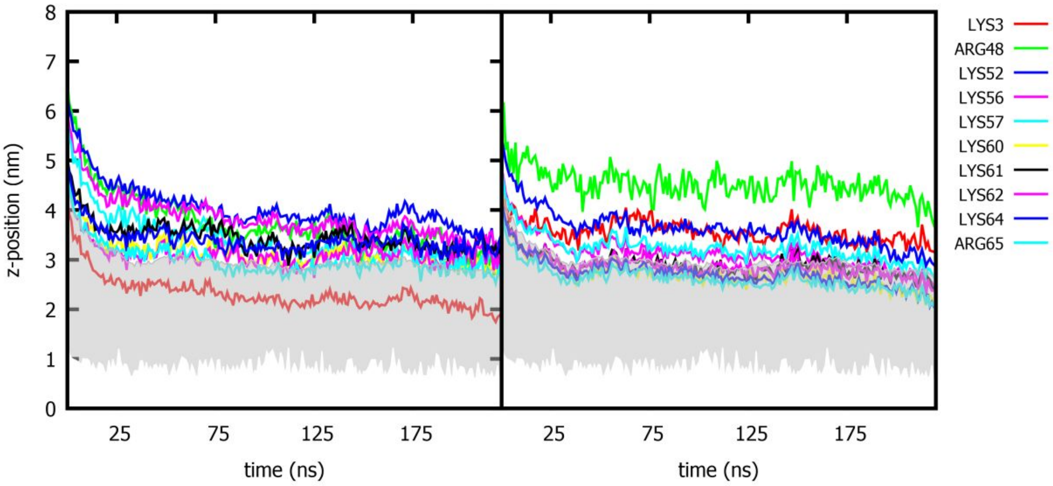

GLUH_310_70. Replica 3

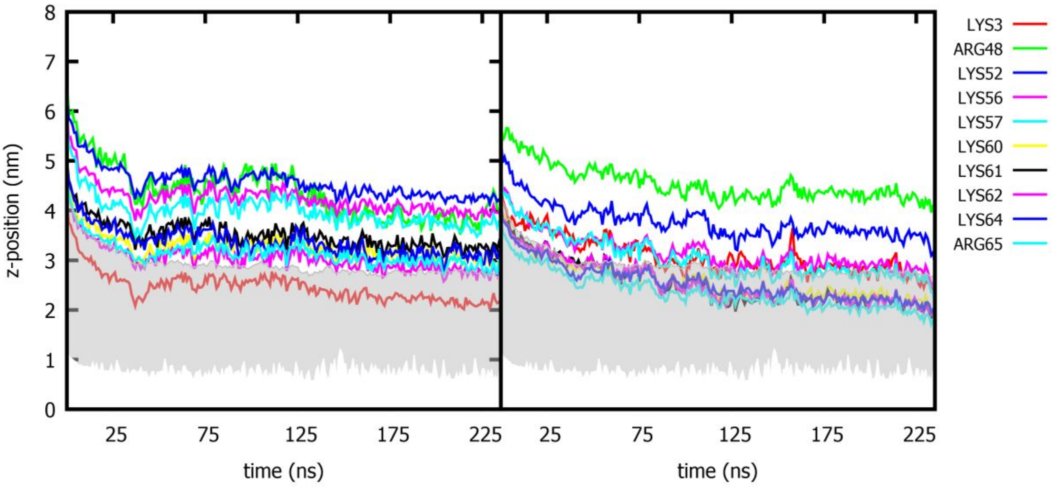

GLUH_330_60. Replica 1

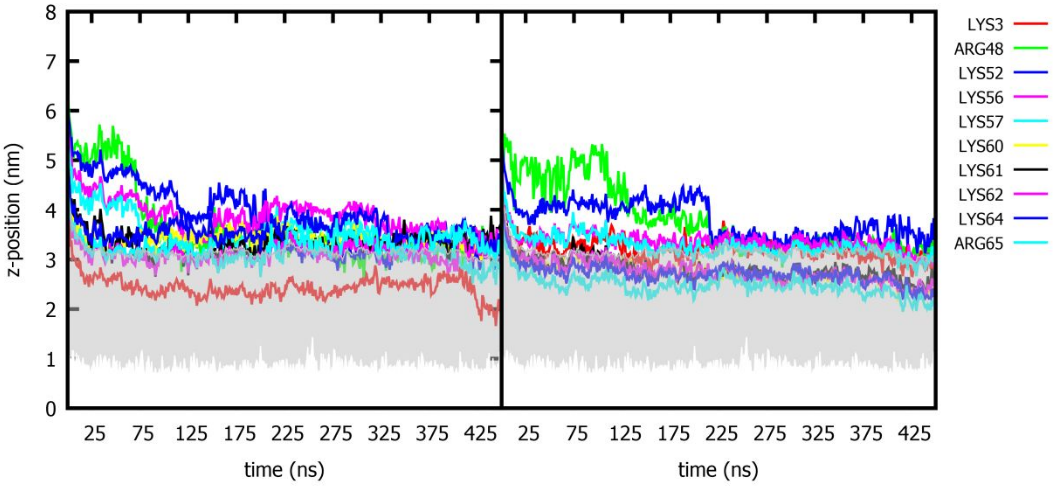

GLUH_330_60. Replica 2

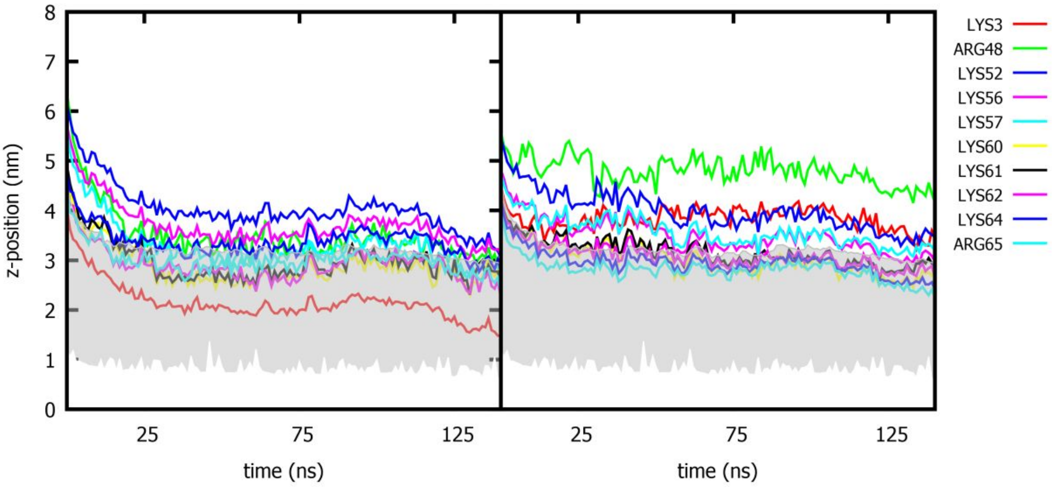

GLUH_330_60. Replica 3

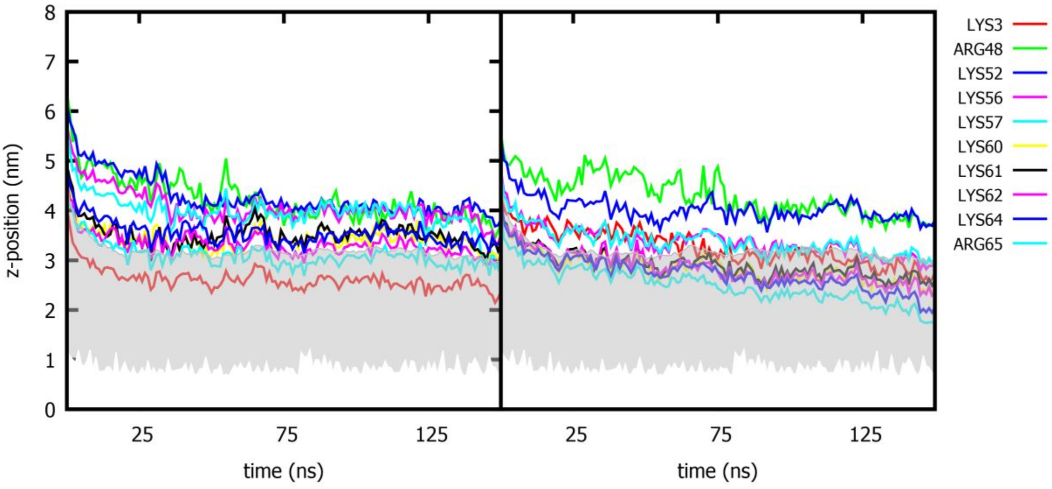

Figure S5. Plots describing the time evolution of the number of H-bonds between each AS-48 protomer and the water molecules.

GLU_310_70 Replica 1

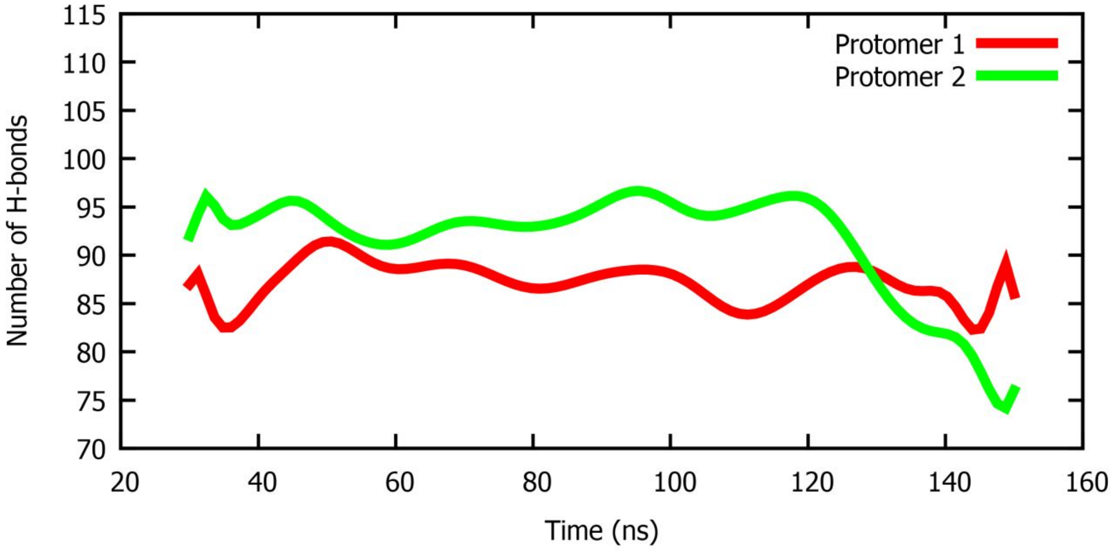

GLU_310_70 Replica 2

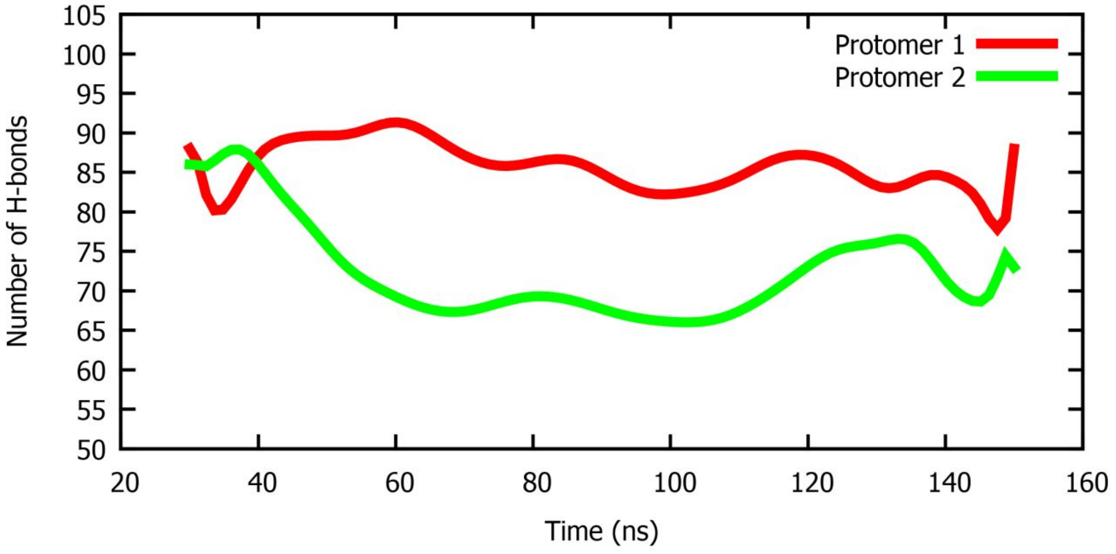

GLU_310_70 Replica 3

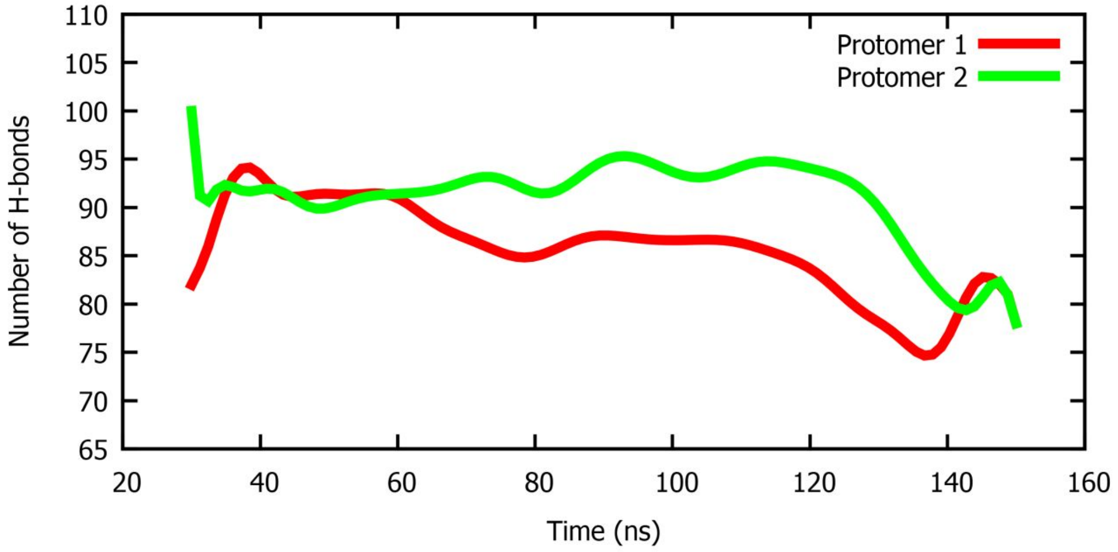

GLU_330_60 Replica 1

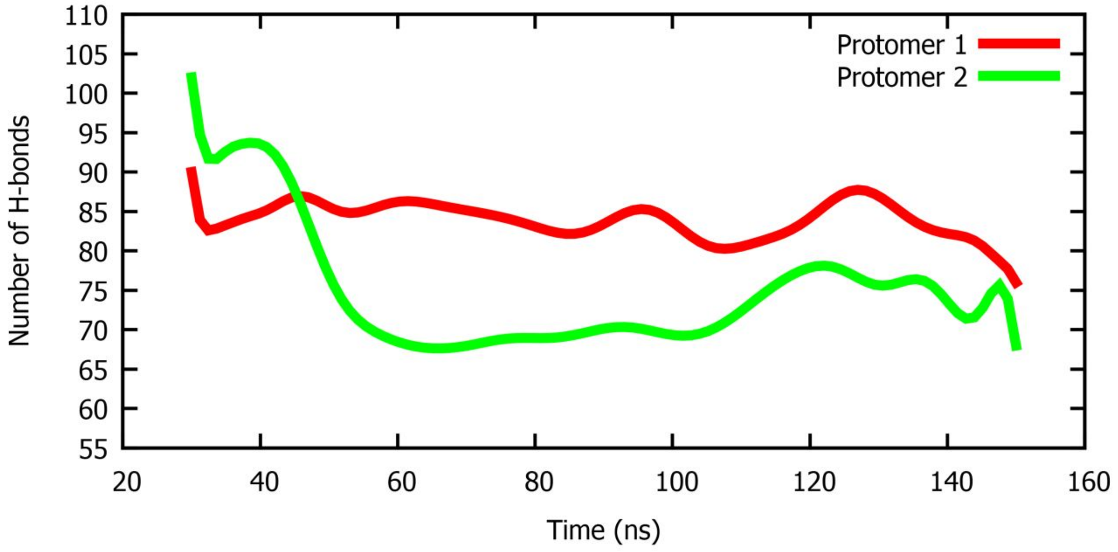

GLU_330_60 Replica 2

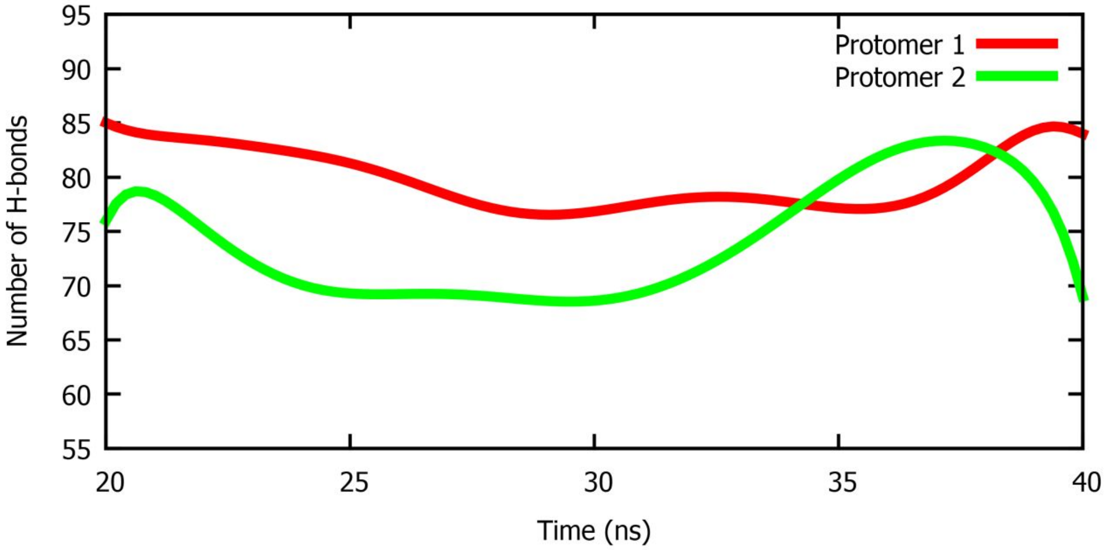

GLU_330_60 Replica 3

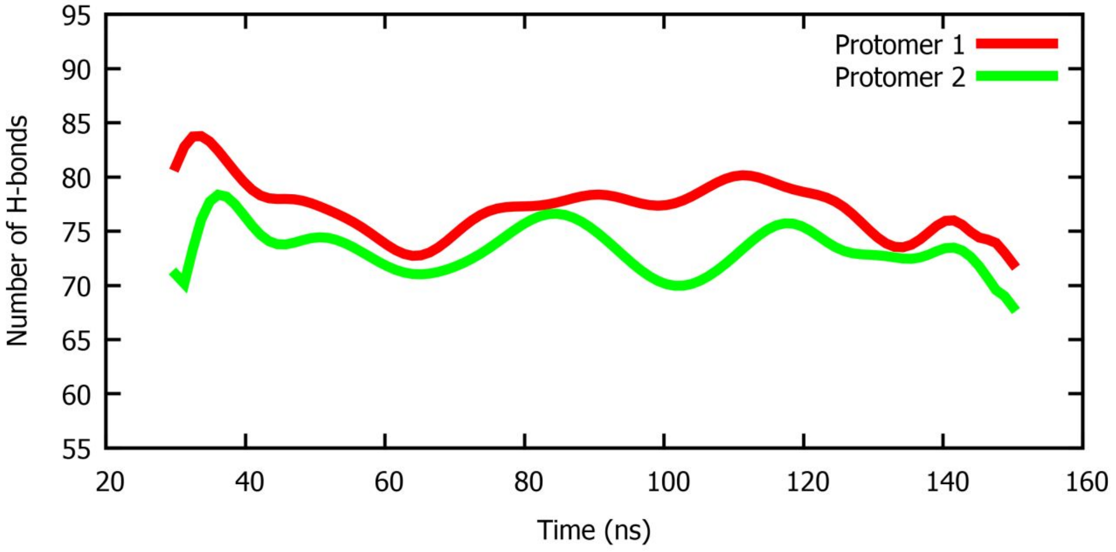

GLUH_310_70 Replica 1

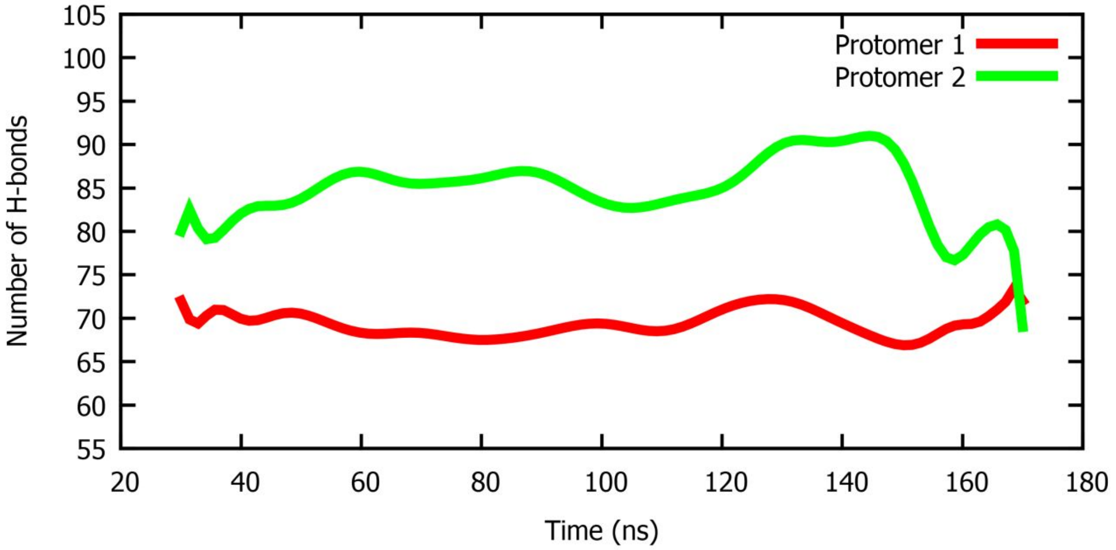

GLUH_310_70 Replica 2

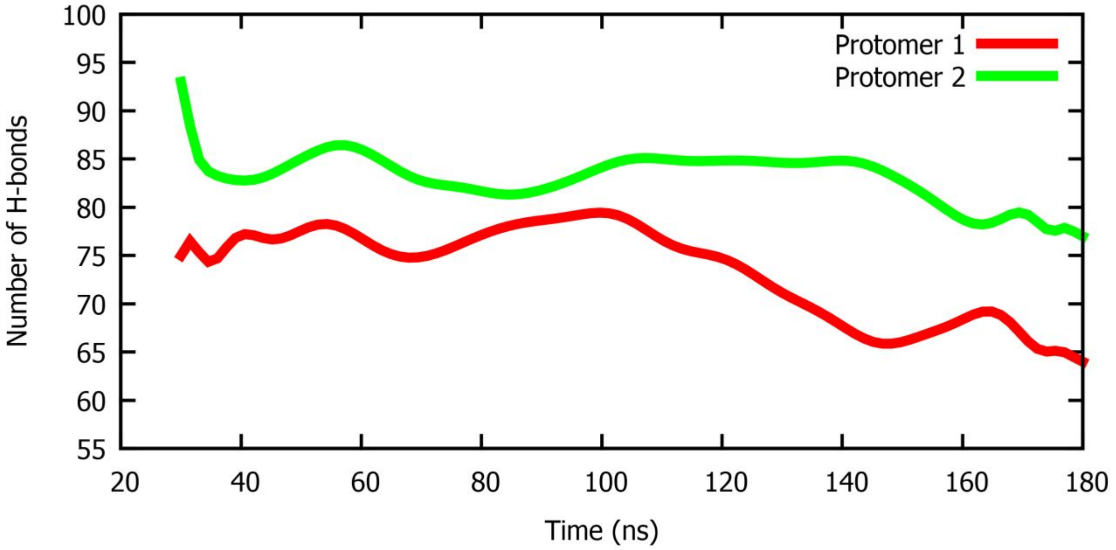

GLUH_310_70 Replica 3

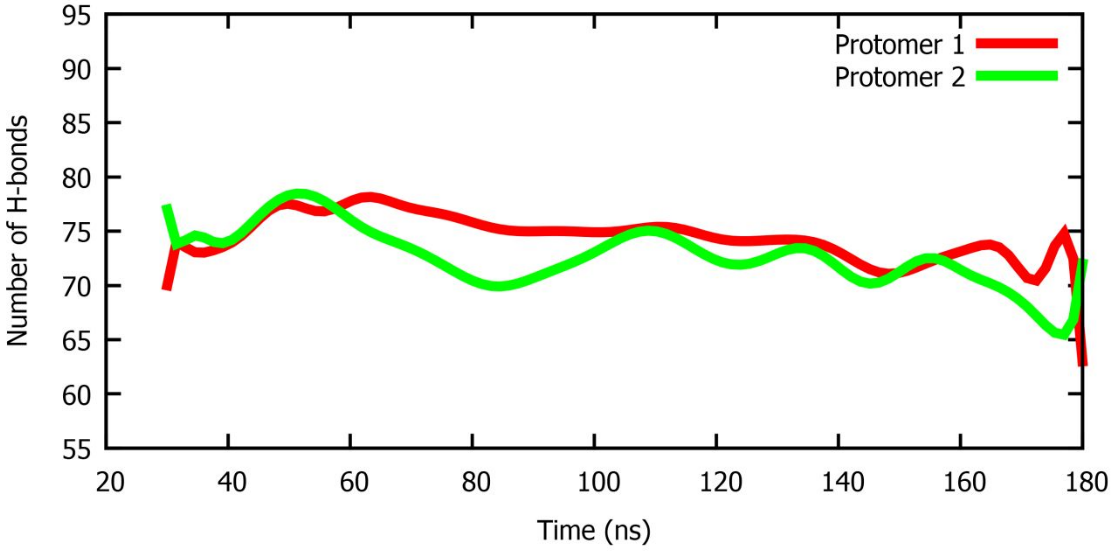

GLUH_330_60 Replica 1

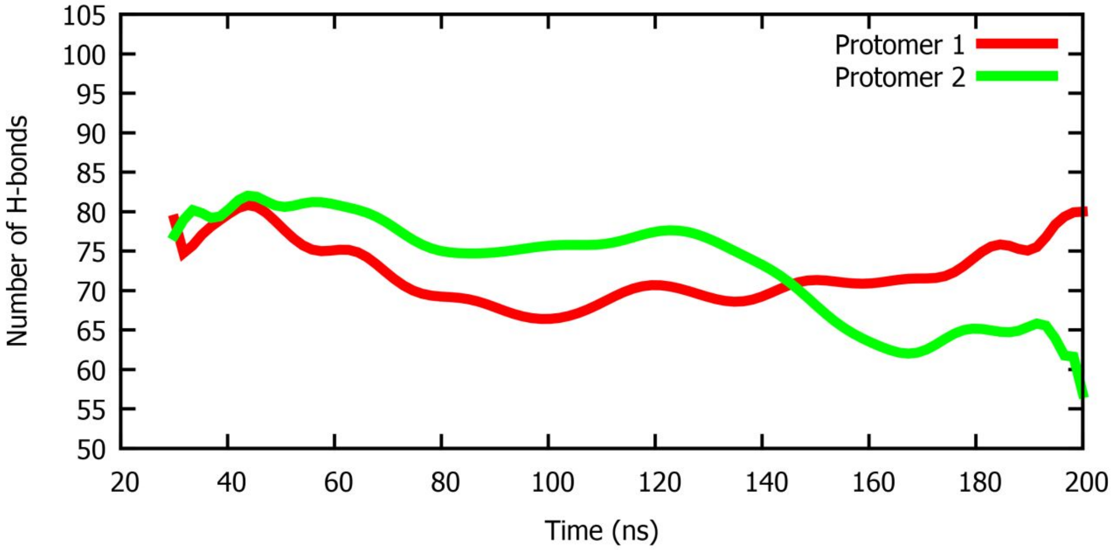

GLUH_330_60 Replica 2

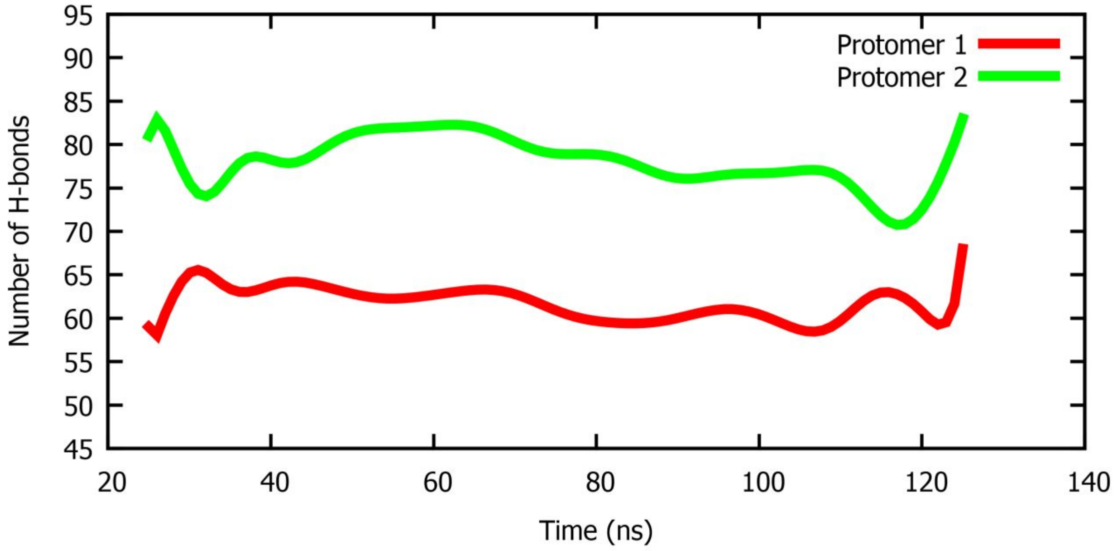

GLUH_330_60 Replica 3

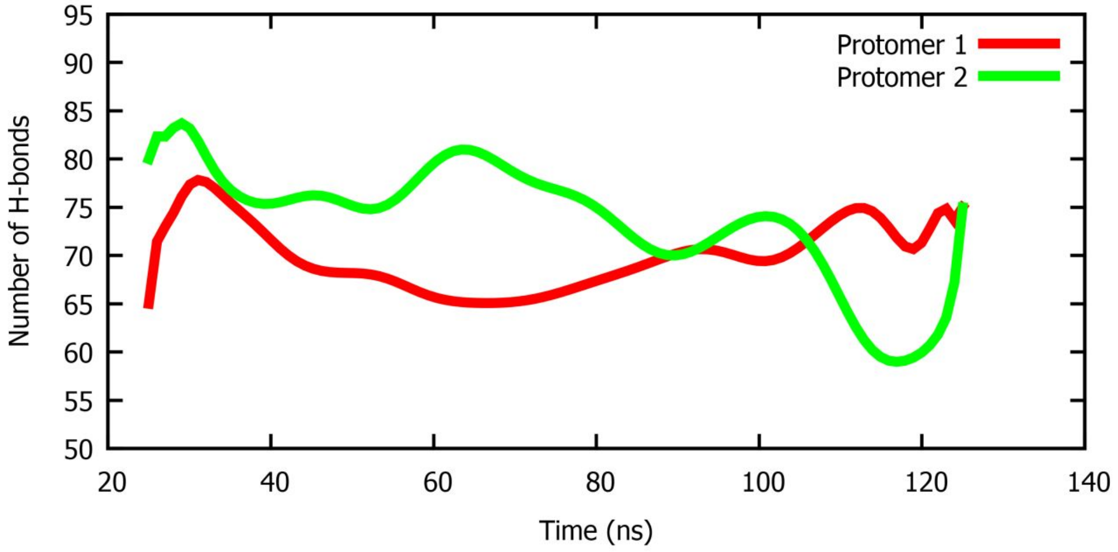

Figure S6. Plots describing the time evolution of the COM corresponding to TRP24 residues along with the distance between those amino acids. The transparent gray area shows the approximate size of the upper bilayer leaflet limited by the average position of the P atoms.

GLU_310_70. Replica 1

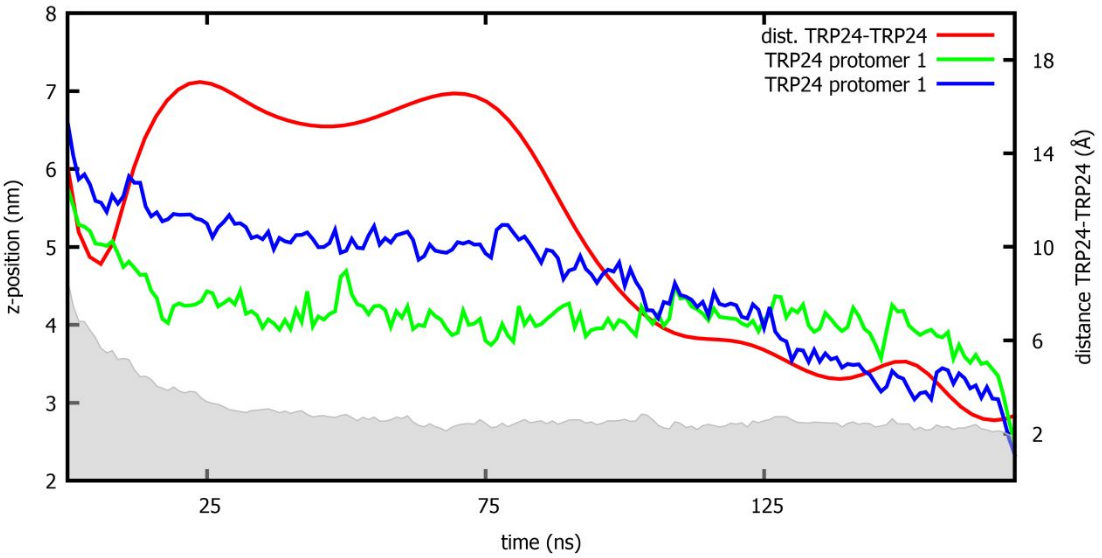

GLU_310_70. Replica 2

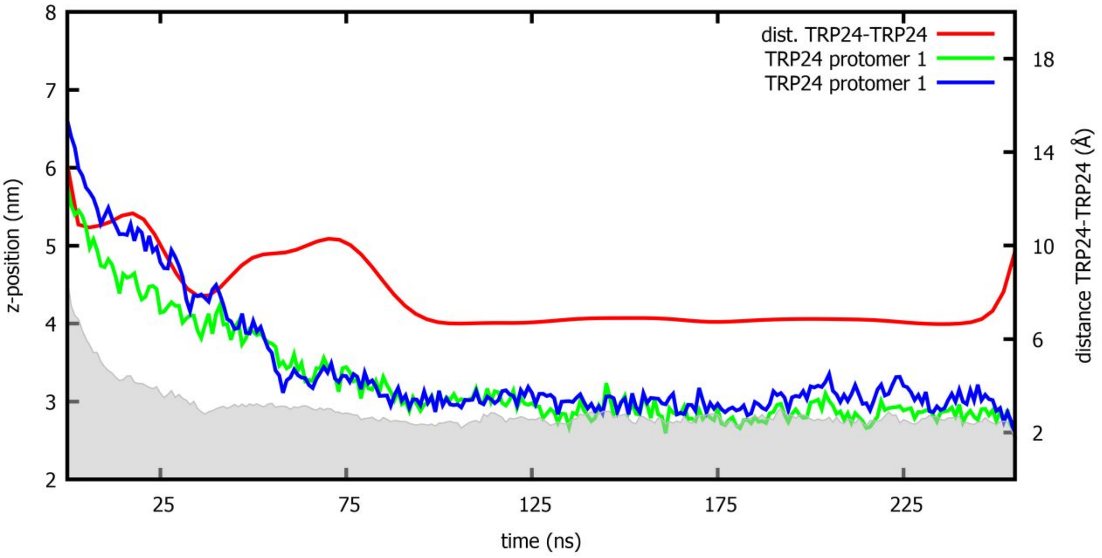

GLU_310_70. Replica 3

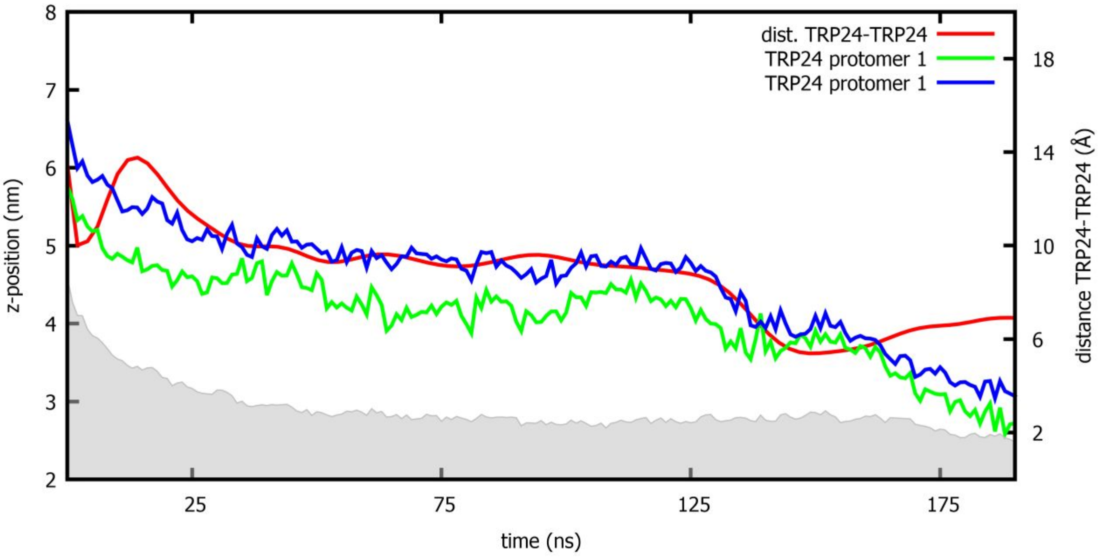

GLU_330_60. Replica 1

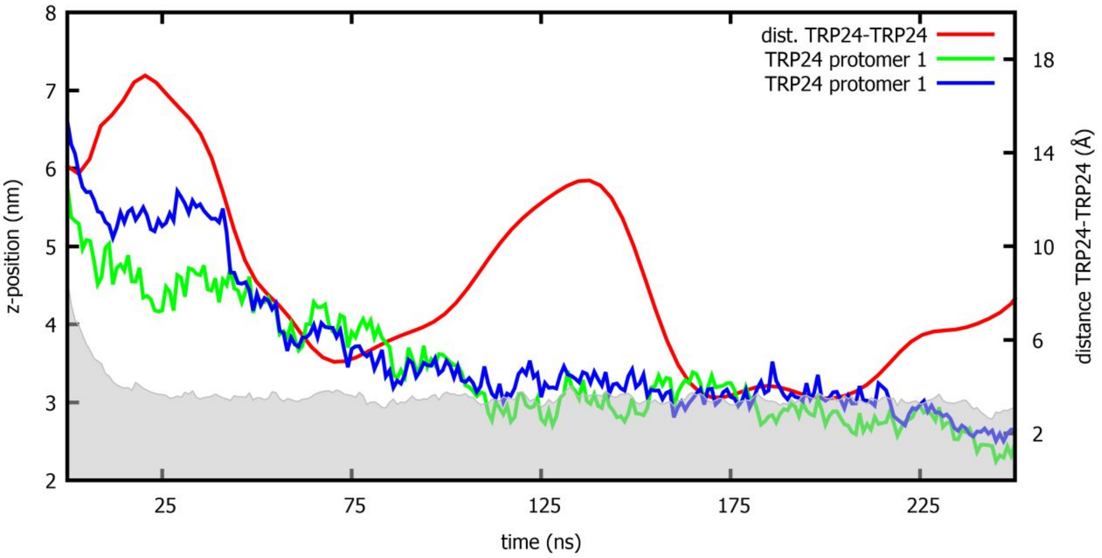

GLU_330_60. Replica 2

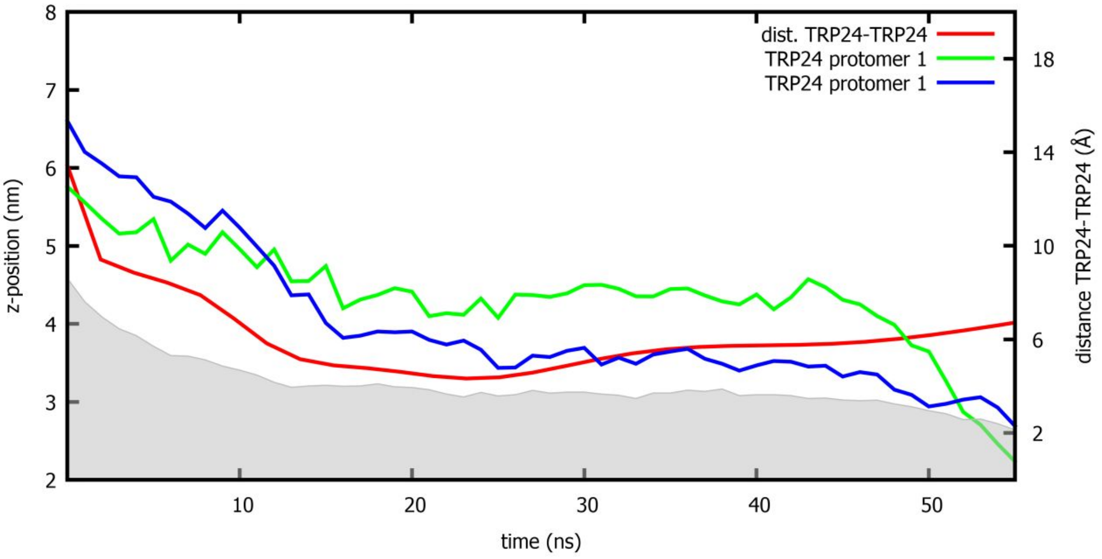

GLU_330_60. Replica 3

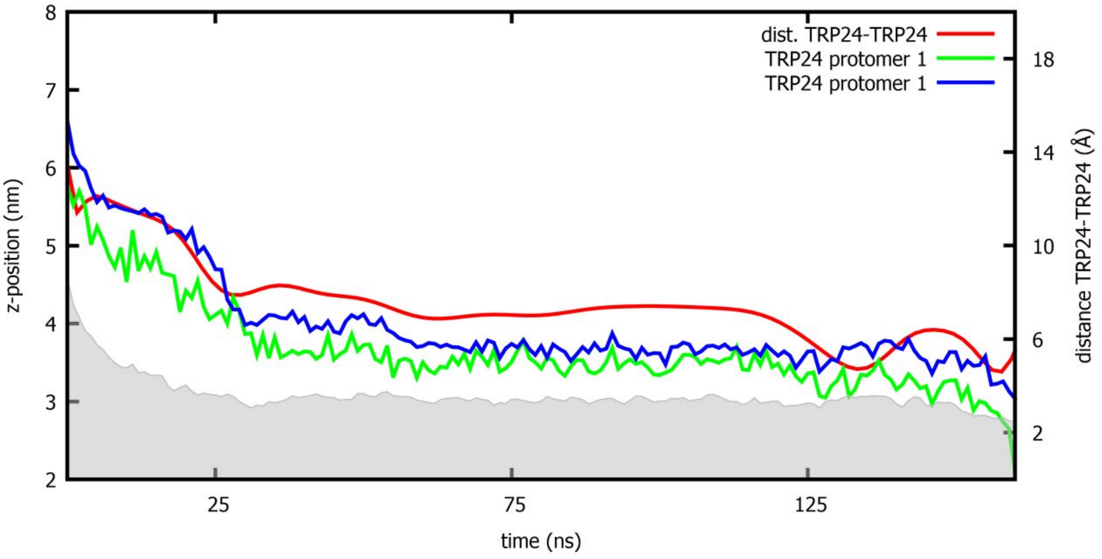

Supplement: Supplementary file 1 — ci1c00838_si_001.pdf [file ci1c00838_si_001.pdf]
